# The Airway Transcriptome in Type 2 Cytokine Biomarker‐High and ‐Low Severe Asthma

**DOI:** 10.1111/all.70441

**Published:** 2026-07-20

**Authors:** Jiashu Shen, Rekha Chaudhuri, Stephen Bicknell, Adel H. Mansur, Rahul Shrimanker, Ian D. Pavord, Stephen J. Fowler, Vanessa Brown, Lorcan P. McGarvey, Peter H. Howarth, Sven‐Erik Dahlén, Ian M. Adcock, Nazanin Zounemat‐Kermani, Joseph R. Arron, Liam G. Heaney, David F. Choy, Timothy S. C. Hinks, Emanuele Marchi, Peter Bradding, the UK Medical Research Council Refractory Asthma Stratification programme (RASP‐UK)

**Affiliations:** ^1^ NIHR Oxford Respiratory BRC, Nuffield Department of Medicine University of Oxford Oxford UK; ^2^ Gartnavel General Hospital, Glasgow, and Institute of Infection& Immunity University of Glasgow Glasgow UK; ^3^ University of Birmingham and Heartlands Hospital, University Hospitals Birmingham NHS Foundation Trust Birmingham UK; ^4^ School of Biological Sciences, Faculty of Biology, Medicine and Health University of Manchester, Manchester Academic Health Science Centre and NIHR Manchester Biomedical Research Centre, Manchester University Hospitals NHS Foundation Trust Manchester UK; ^5^ Wellcome‐Wolfson‐ Centre for Experimental Medicine Queen's University Belfast School of Medicine, Dentistry and Biomedical Sciences Belfast UK; ^6^ School of Clinical and Experimental Sciences University of Southampton, NIHR Southampton Biomedical Research Centre Southampton UK; ^7^ Department of Medicine, Huddinge, and Institute of Environmental Medicine, Karolinska Institutet Stockholm Sweden; ^8^ Centre for Allergy Research Karolinska Institutet Stockholm Sweden; ^9^ Department of Respiratory Medicine and Allergy Karolinska University Hospital Stockholm Sweden; ^10^ National Heart and Lung Institute, Imperial College London UK; ^11^ Data Science Institute, Imperial College London London UK; ^12^ Genentech, Inc South San Francisco California USA; ^13^ Department of Respiratory Sciences, University of Leicester NIHR Leicester Biomedical Research Centre, Glenfield Hospital Leicester UK

**Keywords:** corticosteroid resistance, mechanisms, remodelling, severe asthma, transcriptome

## Abstract

Severe asthma is a heterogeneous disease. The mechanisms driving airway pathology when type 2 (T2) cytokine activity is suppressed remain poorly understood. This study aimed to provide insight by identifying the airway molecular pathways of T2 biomarker‐high and ‐low severe asthma. We analysed clinical and transcriptomic data from bronchial biopsies and brushes in the UK Refractory Asthma Stratification Programme multi‐centre severe asthma cohort (18 corticosteroid‐resistant T2 biomarker‐high [T2‐high], 23 T2 biomarker‐intermediate [T2‐intermediate], 11 T2 biomarker‐low [T2‐low]) plus 20 healthy controls pre‐ and post‐treatment with high‐dose inhaled corticosteroids (ICS). Many genes dysregulated in asthma vs. health were concordantly dysregulated in healthy subjects receiving ICS. Severe asthma as a whole, independent of confounding by ICS, was characterised by upregulation of mucins, *CEACAM5*, typical T2‐genes (*POSTN*, *CLCA1, CCL26*), epithelial mast cell genes, and *CPA4*. T2‐high severe asthma demonstrated upregulated T2‐dependent genes, epithelial barrier and keratin genes, adaptive immune responses, and impaired ciliary function. T2‐low asthma showed upregulated Th1‐ and IL‐17‐associated genes (*IDO1*, *CXCL10*, *GBP1*, *LAG3*), interferon‐γ signalling, neuroimmune pathways, airway smooth muscle‐related genes, and neutrophil enrichment. T2‐intermediate asthma exhibited a mixed molecular profile sharing features of T2‐high and T2‐low endotypes, with selective expression of the pathogen defence and antiviral response genes. The results were validated using bronchoscopy data from the U‐BIOPRED Consortium. This study defines airway molecular endotypes of severe asthma associated with T2 biomarker high and low phenotypes, independent of corticosteroid effects. These findings offer insights for severe asthma management and the development of targeted biologic therapies.

## Graphical abstract

This study distinguishes severe asthma–related airway transcriptomic changes from the effects of high‐dose ICS. Most dysregulated genes in severe asthma show concordance with corticosteroid responses. Corticosteroid‐resistant T2‐high asthma featured T2‐high and epithelial barrier gene upregulation, while T2‐low asthma featured Th1/IL‐17 signatures. ASM, airway smooth muscle; ICS, inhaled corticosteroid; IL, interleukin; Th, T helper cell.

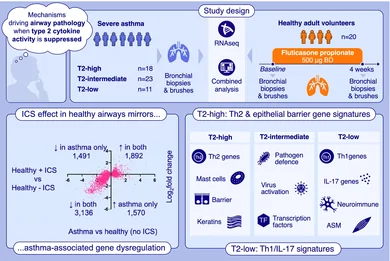

**Abbreviations**
ACQ: asthma control questionnaire
AHR: airway hyperresponsiveness
ASM: airway smooth muscle
BDP eq: beclometasone dipropionate equivalent
BTS: British Thoracic Society
DEG: differentially expressed genes
FDR: false discovery rate
FeNO: fractional exhaled nitric oxide
FER: forced expiratory ratio
FEV_1_
: forced expiratory volume
FVC: forced vital capacity
GEO: Gene Expression Omnibus
GO: gene ontology
GSEA: gene set enrichment analysis
GSVA: gene set variation analysis
HVS: Leicester Healthy Volunteer Study
ICS: inhaled corticosteroid
IL: interleukin
IQR: interquartile range
Log2FC: log2 fold change
PC_20_
: provocative concentration causing 20% fall in FEV_1_
RASP: United Kingdom Refractory Asthma Stratification Programme
REC: Research Ethics Committee
RPA: rank product analysis
SEM: standard error mean
T2: type 2 cytokines (IL‐4, IL‐5, IL‐13)
T2‐high: T2 biomarker‐high FeNO non‐suppressor
T2‐intermediate: T2 biomarker‐intermediate
T2‐low: T2 biomarker‐low
TF: transcription factor
VST: variance stabilising transformation
WGCNA: weighted gene co‐expression network analysis

## 1 Introduction

Asthma affects 260–350 million people [1]. Approximately 10% show resistance to current therapies and incur substantially greater asthma health care costs [2]. Asthma is a heterogeneous disease characterised by airway hyperresponsiveness (AHR), variable airflow obstruction, airway inflammation, mucus hypersecretion and airway remodelling with epithelial dysfunction, mucous gland hyperplasia, and increased airway smooth muscle (ASM) mass. The relationship between these elements and molecular pathways is poorly understood, as are the pathways underlying distinct clinical phenotypes.

Blood or airway eosinophilia with a raised fraction of exhaled nitric oxide (FeNO) is found in 50%–80% of people with mild corticosteroid‐naïve asthma [3, 4]. This eosinophilic phenotype is associated with an airway gene expression signature driven by interleukin (IL)‐4 and IL‐13 [5, 6, 7, 8], with tissue eosinophilia dependent also on IL‐5 [9]. Together, these cytokines constitute the type 2 (T2) inflammatory pathway. Monoclonal antibodies targeting IL‐4/−13 (via IL‐4Rα), IL‐5/IL‐5Rα, or the epithelial alarmin TSLP, are most effective in the T2‐high group [10, 11].

Severe asthma that is not T2‐high responds poorly to both inhaled corticosteroids (ICS) and T2 biologics and is not understood at a molecular level. Transcriptomic analyses from airway biopsies have demonstrated mutually exclusive expression of a typical T2 cytokine‐dependent gene signature and an IL‐17‐dependent gene signature in asthma [7, 8]. However, 50% of uncontrolled severe asthma patients receiving high‐dose ICS are neither T2‐high nor IL‐17‐high [7, 8]. The molecular mechanisms underlying these heterogeneous clinical phenotypes and their associated pathological features remain to be explored.

Three specific obstacles related to treatment confound mechanistic studies in asthma. Firstly, inadequate adherence is a common feature confounding the assessment of many people with difficult asthma [12]. Secondly, symptom‐directed treatment can lead to inappropriately high use of ICS in those with T2‐intermediate or ‐low disease, obfuscating accurate clinical phenotyping [13]. Thirdly, mechanistic studies tend to use healthy control subjects not receiving ICS as comparator groups, making it impossible to distinguish molecular signatures attributable to the immunopathology of asthma from those consequent upon therapeutic ICS.

To address these challenges, several complementary strategies were incorporated into the study design. First, we collected samples from participants with objectively verified treatment adherence, including the use of FeNO‐suppression testing, which differentiates between corticosteroid‐sensitive T2‐high disease, typically associated with inadequate adherence, and true corticosteroid‐resistant T2‐high disease [14]. Second, we included patients completing a clinical trial in which therapeutic corticosteroid use was optimised by a biomarker‐driven protocol [15]. Third, to disentangle asthma‐related transcriptional changes from corticosteroid‐induced effects, a comparator group of healthy participants treated with high‐dose ICS was included [16].

This study, therefore, aimed to characterise the airway transcriptome of severe asthma without ICS confounding, and explore this at the extremes of the T2 biomarker spectrum to define the key features that contribute to corticosteroid‐resistant T2‐high, T2‐intermediate and T2‐low severe asthma. The immunohistological features and sputum cytokine profiles of this severe asthma cohort have been published previously [17].

## 2 Methods

### 2.1 Study Population

All participants gave written informed consent. The prospective multi‐centre United Kingdom Refractory Asthma Stratification Programme (RASP) bronchoscopy study was approved by the East Midlands—Leicester South Research Ethics Committee (REC) (reference 16/EM/0260) and registered at ClinicalTrials.gov: NCT02883530. It recruited 52 patients with severe asthma; 18 T2‐high, 23 T2‐intermediate, and 11 T2‐low [17]. Clinical data were collected, and bronchoscopies were performed. People with severe asthma aged 18–70 were eligible, were current non‐smokers with a < 15 pack‐year history, and were not receiving biologics. Recruitment was prospective using pre‐defined inclusion and exclusion criteria [17] as follows: (i) patients with a previous FeNO ≥ 45 ppb and blood eosinophils ≥ 0.3 × 10^9^/L, who did not suppress their FeNO during a FeNO suppression test [14, 18, 19] during routine clinical care (T2‐high); (ii) patients with a FeNO ≤ 30 ppb and blood eosinophils ≤ 0.2 × 10^9^/L identified in clinic (T2 low); (iii) patients who had exited the RASP T2‐biomarker (FeNO, blood eosinophils, periostin)‐driven treatment optimisation study [15] with intermediate or low biomarker measurements (T2‐intermediate, or T2 low as described above).

The Leicester Healthy Volunteer Study (HVS) (NCT: 2476825; REC approval 15/EM/0313) included 20 healthy participants receiving maximum licenced dose inhaled fluticasone propionate 500 mcg bd daily (equivalent to 2000 mcg daily beclometasone) for 4 weeks [16]. Bronchoscopies were performed before and after 4 weeks of ICS therapy. Biopsy and brush samples in RASP and HVS were collected from 2nd‐5th generation bronchi under direct vision using the same standard operating procedures; both studies ran contemporaneously.

The study is summarised in Figure S1.

For validation, we used the previously described U‐BIOPRED cohort, classifying their T2 status using the above biomarker thresholds (see supplementary methods).

### 2.2 RNA Sequencing and Bioinformatics Analysis

Full details are provided in the supplement. Bulk RNA sequencing was performed on bronchial biopsies and brushings (Figure S1 summarises sequencing batches). The RNA Sequencing data are deposited in the Gene Expression Omnibus (GEO) (accession numbers GSE242048, GSE301357). Differential expression analysis was performed using the *DESeq2* package [20] with Benjamini–Hochberg false discovery rate (FDR) correction < 0.05 considered significant, adjusted for age, sex and previous smoking status. To identify gene signatures associated with inflammatory biomarkers, we performed differential gene expression analyses in which each gene's expression was modelled as a continuous linear predictor of biomarker levels.

Data integration with the Leicester HVS to explore the ICS effect on asthma gene transcription was performed initially using ComBat from the *sva* package to remove batch effects [21]. To confirm robustness against batch effects and variability, differential expression was also assessed using the non‐parametric, rank‐based method implemented in the *RankProd* package [22]. We also performed Gene Ontology (GO) enrichment analysis [23], Gene Set Enrichment Analysis (GSEA) [24] to investigate pathway‐level changes across T2‐phenotypes [25], Gene Set Variation Analysis (GSVA) [26] to evaluate the enrichment of gene signatures including T2 cytokine‐ and IL‐17‐dependent gene signatures [7, 8] across T2 phenotypes, and Weighted Gene Co‐expression Network Analysis (WGCNA) [27] to identify T2 phenotype‐associated modules. Transcription factor (TF) activity was inferred using the DoRothEA regulon database [28]. For unsupervised clustering of the gene expression matrix, K‐means clustering was applied using the *ClusterGVis* package [29]. Reference‐based cell deconvolution was conducted using Travaglini [30] to estimate relative proportions of cell subsets in each sample.

### 2.3 Statistical Analysis

Please see the Supporting Information. For assessment of differential gene expression, a DESeq2 log2 fold change (log2FC) ± ≥ 0.5 was considered biologically relevant. All statistical analyses were performed using R software (version 4.4.0), with significance set at *p* < 0.05.

## 3 Results

### 3.1 Clinical Characteristics

The clinical characteristics of the pre‐defined RASP bronchoscopy study groups are summarised in Table 1. As reported previously [17], both FeNO and blood eosinophils were markedly increased in T2‐high asthma compared to the other groups, as expected from the recruitment criteria. Clinical correlations are presented in the supplementary results (Figure S2).

**TABLE 1:** Participant characteristics.

Characteristics	T2‐high (*n* = 18)	T2‐intermediate (*n* = 23)	T2‐low (*n* = 11)	Healthy controls (*n* = 20)	*p*
Demographics					
Age, years	56 [48–62]**	56 [46–63]**	53 [33–56]	37.5 [22–52.3]	< 0.001
Sex, Females, %	9 (50%)	11 (48%)	4 (36%)	12 (60%)	0.65
Ethnicity, Caucasian, %	18 (100%)	20 (87%)	11 (100%)	16 (80%)	0.12
BMI, kg/m^2^	29.8 [24.9–33.9]**	31.4 [26.4–39.8]***	29.2 [27.6–32.6]*	23.8 [21.3–27.8]	< 0.001
Ex‐smoker, %	5 (28%)	7 (30%)	2 (18%)	4 (20%)	0.8
Symptoms and history					
Age onset, years	28.2 ± 18.8	27.1 ± 20.5	19.6 ± 17.5	*N*/A	0.47
Asthma duration, years	26.6 ± 19.2	26.4 ± 15.2	26.3 ± 14.0	*N*/A	1
Atopic^a^	81%	62%	73%	25%	0.001
ACQ‐5	1.7 [0.8–2.5]	1.2 [0.0–2.0]	1.8 [0.6–2.4]	*N*/A	0.49
Annual severe exacerbations	2 [1.0–3.3]	1 [0.0–2.0]	4 [0.0–6.0]	*N*/A	0.042
Annual A&E attendances	0 [0.0–1.1]	0 [0.0–0.0]	0 [0.0–4.0]^#^	*N*/A	0.012
Medications					
ICS, %	18 (100%)	23 (100%)	11 (100%)	*N*/A	*N*/A
ICS dose, mcg BDP eq	2,000 [2,000–2,000]	2,000 [1,000–2,000]	2,000 [2,000–2,000]	*N*/A	0.75
Maintenance OCS, %	6 (33%)	8 (35%)	4 (36%)	*N*/A	1
OCS dose, mg	10 [5.0–12.5]	8.8 [5.0–17.5]	8.8 [5.6–10.0]	*N*/A	0.93
LABA, %	18 (100%)	23 (100%)	11 (100%)	*N*/A	*N*/A
LAMA, %	9 (50%)	9 (39%)	7 (64%)	*N*/A	0.3
LTRA, %	10 (56%)	11 (48%)	1 (9%)	*N*/A	0.03
Lung function					
FEV_1_ pre‐bronchodilator, L	2.04 [1.67–2.78]***	2.43 [1.99–2.80]**	2.83 [2.18–3.31]	3.46 [2.78–3.86]	< 0.001
FEV_1_ pre‐bronchodilator, % pred	67.5 [55.8–80.5]***	77.5 [71.7–93.3]***	86.3 [66.3–99.3]	102.5 [92.5–110.8]	< 0.001
FEV_1_/FVC pre‐bronchodilator, %	59.8 [55.9–71.1]***	68.8 [62.6–76.8]***	70.3 [57.0–81.6]*	82.5 [80.0–85.8]	< 0.001
Biomarkers					
IgE, kU/L	129 [70–260]	90 [18–325]	231 [85–303]	Not done	0.44
FeNO, ppb	71.5 [42.5–103.3]***^,###,§§^	25 [16.0–36.5]	15 [10.0–18.0]	18.0 [11.3–25.0]	< 0.001
Blood eosinophils, ×10^9^/L	0.37 [0.23–0.57]***^,##,§§§^	0.17 [0.06–0.3]	0.07 [0.03–0.15]	0.12 [0.06–0.15]	< 0.001
Sputum eosinophils, %	10.7 [2.3–32.8]^§§^	1.5 [0.2–4.2]	0.3 [0.2–0.4]	Not done	0.006
Sputum neutrophils, %	17.1 [2.0–36.2]	40 [8.6–83.5]	68.8 [28.1–3.3]	Not done	0.097

*Note:* Continuous variables are presented as mean ± SD or median [interquartile range]. For continuous variables, the ANOVA or the Kruskal–Wallis test was used across applicable groups. For categorical variables, the Chi‐squared test was used across applicable groups. *p < 0.05, **p < 0.01, ***p < 0.001 compared with healthy control subjects. ^#^ *p* < 0.05, ^##^ *p* < 0.01, ^###^ *p* < 0.001 compared with T2‐intermediate. ^§§^ *p* < 0.01, ^§§§^ *p* < 0.001 compared with T2‐low.

Abbreviations: A&E, Accident and Emergency; ACQ‐5, Asthma Control Questionnaire‐5; BMI, body mass index; FeNO, Fractional concentration of exhaled nitric oxide; FEV1, Forced expiratory volume in 1 s; FVC, Forced vital capacity; ICS, inhaled corticosteroids; Ig, immunoglobulin; IQR, interquartile range; LABA, long‐acting Beta2 agonist; LAMA, long‐acting muscarinic antagonist; LTRA, leukotriene receptor antagonist; OCS, oral corticosteroids; SD, standard deviation; THEO, theophylline.

^a^ Atopic refers to the presence of a positive skin test or the presence of a raised specific IgE to a common aeroallergen.

### 3.2 Differential Gene Expression Analysis in Asthma Vs. Healthy Controls Not Receiving ICS

**FIGURE 1:**
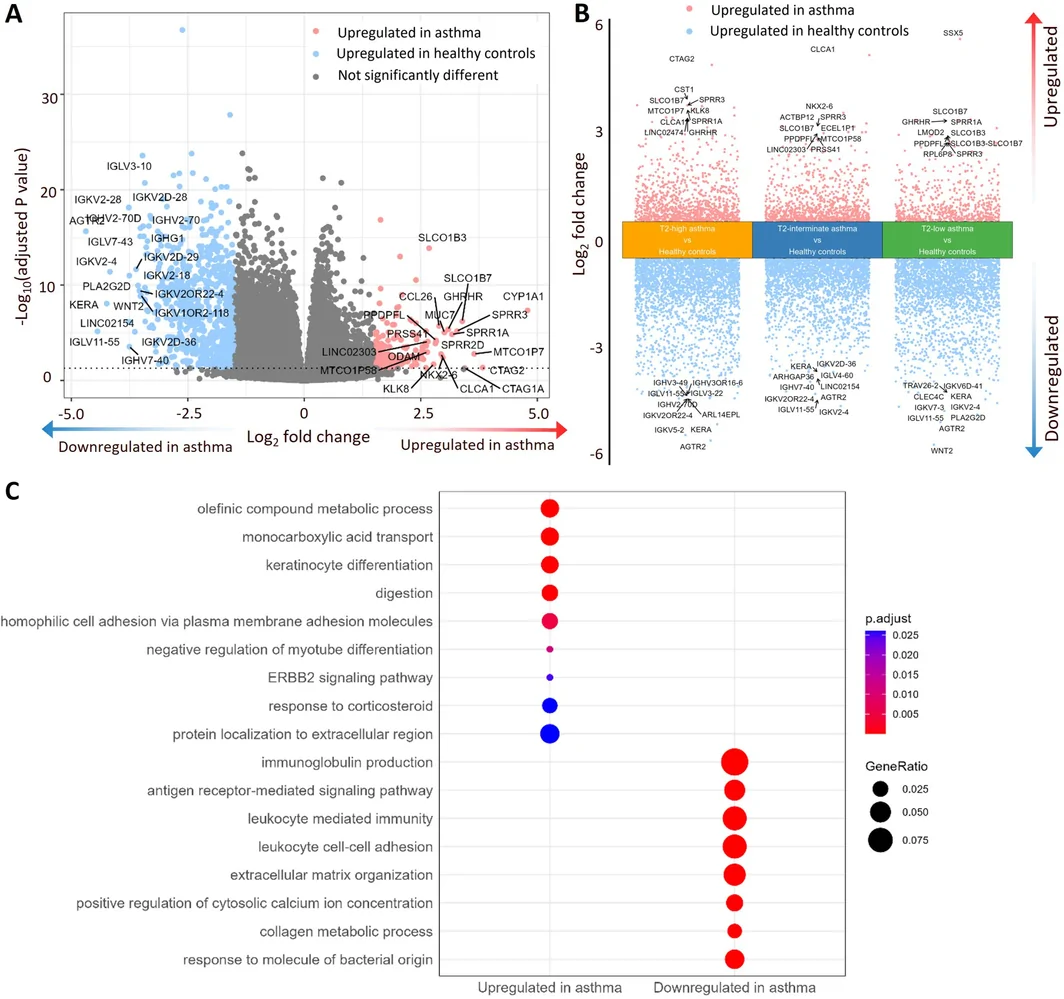
Differential gene expression and pathway enrichment between severe asthma and healthy individuals not receiving inhaled corticosteroids, in bronchial biopsies. (A) Differential gene expression in biopsies between asthma and healthy controls not receiving ICS, adjusted for age, sex, and previous smoking status. Volcano plot from bronchial biopsies with adjusted *p* < 0.05 and log2FC > 0.5. The x‐axis represents the log2FC in gene expression, and the y‐axis indicates the −log10 adjusted *p*‐value. Genes significantly upregulated in asthma are shown in red, while those downregulated in asthma are in blue; non‐significant genes are shown in grey. The top 20 upregulated and top 20 downregulated genes (ranked by log fold change) are labelled. (B) Differential gene expression in biopsies across asthma phenotypes vs. healthy controls not receiving ICS, adjusted for age, sex, and previous smoking status. All displayed genes are significantly differentially expressed (*padj* < 0.05). Each point represents a gene's log2FC, with red indicating log2FC > 0.5, blue indicating log2FC < −0.5, and grey indicating −0.5 ≤ log2FC ≤ 0.5. (C) Gene Ontology (GO) biological process enrichment of genes differentially regulated in biopsies in asthma and healthy controls not receiving ICS. Dot size indicates gene ratio, and dot colour indicates adjusted *p*‐value. GO terms enriched in each group are plotted on the corresponding side of the x‐axis.

We analysed differentially expressed genes (DEGs) in bronchial biopsies between severe asthma patients and healthy individuals not receiving ICS. Most DEGs were downregulated (755 down, 192 up with log2FC ± ≥ 1.5 [Table S1]; and 2922 down and 1269 up with log2FC ± ≥ 0.5 [Figure 1A,B, Table S1]). Asthma was associated with upregulation of classic T2‐driven inflammatory genes, including *CLCA1*, *CCL26*, and *CEACAM5* [31], the gel‐forming mucin *MUC6*, and *AREG* (amphiregulin), associated with epithelial growth and corticosteroid‐resistant mucus production [32]. Genes induced by ICS in healthy airways (*ALOX15, PHACTR3*, *EREG, CYP1A1*) [16] were also upregulated.

Among downregulated genes in biopsies, 269 were immunoglobulin genes, consistent with ICS treatment [16] (Table S1). Several genes were consistently downregulated in both biopsies and brushings, including *WNT2*, *KIR2DL4*, *AGTR2, GABRG1*, and *CNTNAP5*.

DEGs from bronchial brushings are shown in Table S1. In bronchial brushings, the predominant signal was of transactivation: 214 genes were upregulated, 72 were significantly downregulated with log2FC ± ≥ 1.5 [Table S1]; and 1485 upregulated and 498 downregulated with log2FC ± ≥ 0.5 [Table S1]. Upregulated genes included type 2‐associated cystatins (*CST1/2/4*) [31], *FETUB*, *PPBP* (a neutrophil chemoattractant), *CLC*, defensins (*DEFA1B/A1/A3*), and chemokines (*CCL7/13/24*). The significant DEGs in brushes with log_2_FC > ±0.5 correlated positively with the respective genes in brushes, *r* = 0.43, *p* = 5.17e‐88.

GO analysis of bronchial biopsies indicated enrichment of pathways related to epithelial barrier dysfunction, mucus hypersecretion, tissue remodelling, and impaired repair, together with suppression of immune pathways consistent with corticosteroid effects (Figure 1C).

### 3.3 Differential Gene Expression in Asthma Adjusted for the Confounding Effect of ICS

To separate asthma‐specific immunopathogenic processes from ICS effects, we compared gene expression between severe asthma patients and healthy controls treated with fluticasone 500 mcg twice daily for 4 weeks. Using DESeq2 with ComBat for batch correction, we identified 993 upregulated and 536 downregulated genes in bronchial biopsies (log2FC ± ≥ 0.5), and 638 upregulated and 404 downregulated genes in brushings (log2FC ± ≥ 0.5) (Table S1). There was substantial overlap—16 of the top 20 upregulated biopsy genes were also elevated in brushings. These included *SYNCRIP* [33], *PINX1*, *CPA4*, *CLDN2*, *B3GNT6* [34], previously noted genes (*CST1–4, CEACAM5, CLCA1, FETUB*), and additional shared upregulated genes (*MUC5AC*, *ITLN1*, *CD1A*).

To confirm these results and to ensure signals were not lost using Combat batch correction, we further assessed DEGs using a non‐parametric rank product analysis (RPA) approach. RPA is robust against noise, highly variable gene expression and batch effects, and is generally more sensitive with higher statistical power for detecting DEGs in these types of datasets. (Table 2, Figure S3). Among the 20 most highly upregulated genes, this confirmed the significance of *MUC5AC, SYNCRIP, and CEACAM5*, as well as demonstrating highly significant upregulation of lysozyme (*LYZ*), periostin (*POSTN*), IL‐33 (*IL33*), MUC2, and integrin β8 (*ITGB8*), which regulates TGFβ activation and airway remodelling.

**TABLE 2:**
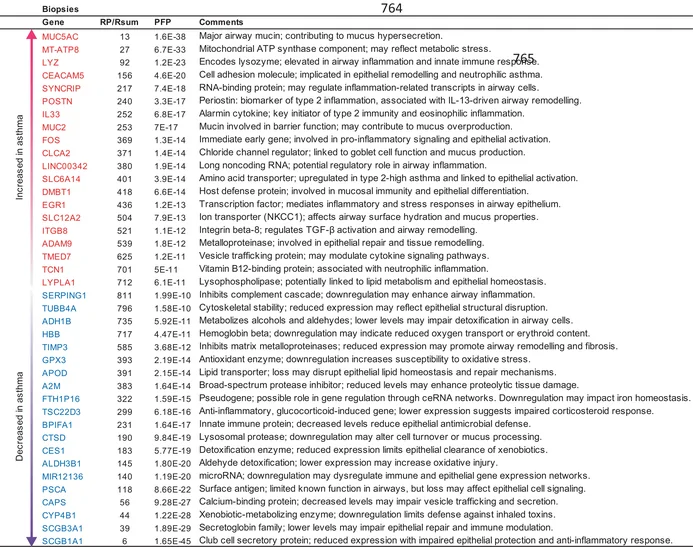
Top differentially expressed genes in bronchial biopsies using a rank product approach comparing all participants with asthma vs. healthy control participants receiving inhaled fluticasone propionate 500 mcg bd daily for 4 weeks. abbreviations: RP/Rsum: rank product/rank sum; PFP: Percentage of false positive predictions.

Strongly downregulated genes in both biopsies and brushes included *SCGB1A1, SCGB3A1, HIF3A*, and *SST* [31, 35, 36]. Of other downregulated genes of interest identified comparing asthma with health (*WNT2*, *KIR2DL4*, *AGTR2, GABRG1*, *CNTNAP5*), only *CNTNAP5* (linked to asthma exacerbations in GWAS) was significantly different to healthy+ICS.

GO analysis of biopsy DEGs in asthma vs. ICS‐treated controls revealed enrichment in adaptive mucosal immunity (Table S2). Downregulated pathways were mainly linked to mitochondrial function and aerobic respiration, associated with anti‐inflammatory immunometabolism. In focused gene GSEA of immune response GO pathways, asthma was associated with pathways related to T cell‐stimulated mast cells, IFNα‐stimulated dendritic cells, virus‐stimulated immune cells, and suppression of pathways related to immunoregulatory T cells (Table S3). Cell deconvolution analysis in biopsies is presented in the online supplement (Figure S4A,B; Table S4).

**FIGURE 2:**
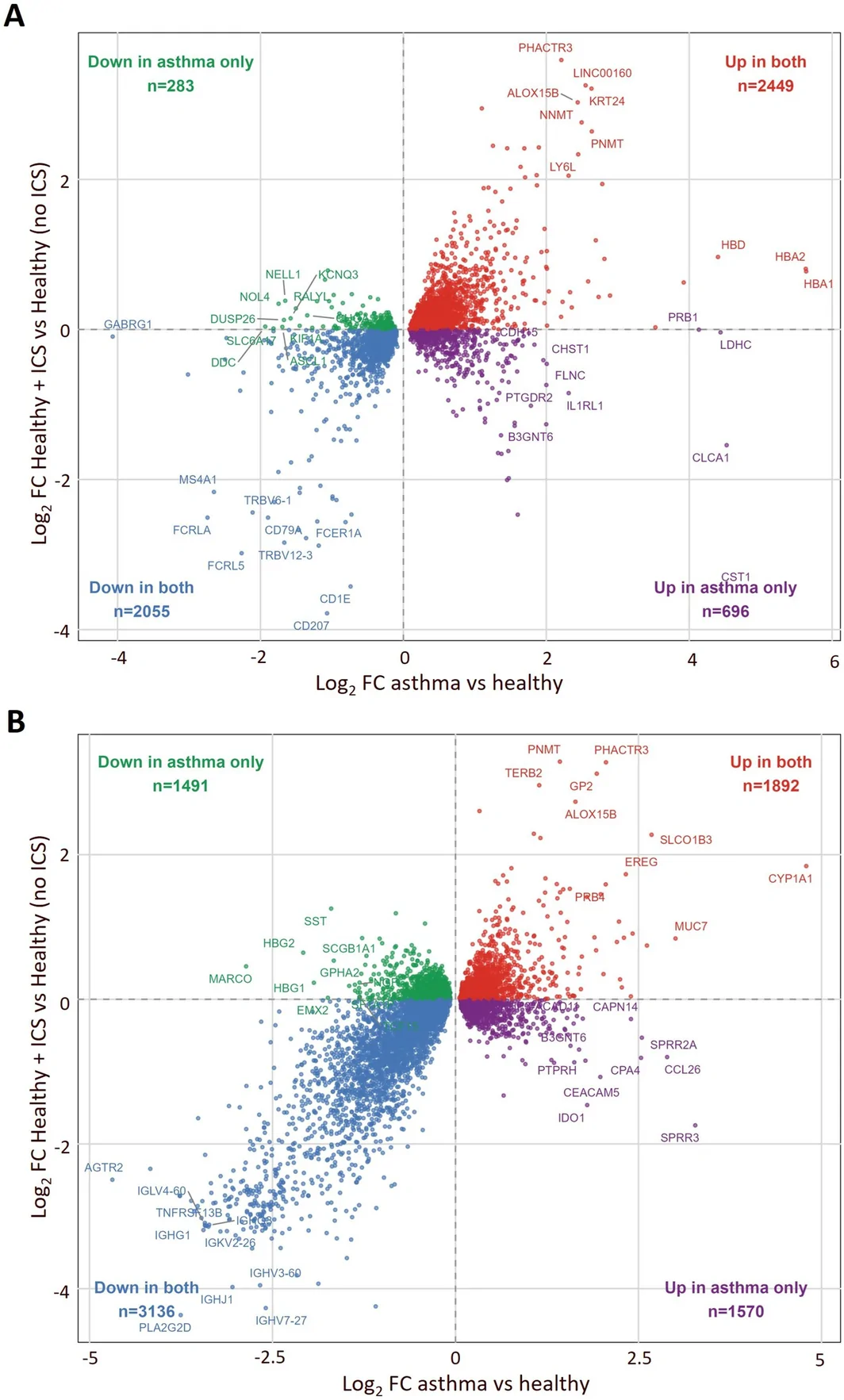
The log2FC in gene expression in asthma vs. healthy (no ICS) plotted against the log2FC in gene expression in healthy+ICS vs. healthy (no ICS), demonstrating asthma‐specific DEGs (discordant genes) and those potentially driven by ICS (concordant genes). (A) biopsies, (B) brushes.

To highlight asthma DEGs which are discordant with ICS effect, i.e., upregulated in asthma vs. health but downregulated or unaltered by ICS in health and vice‐versa, we plotted the log2FC of DEGs identified in asthma compared to health (no ICS) against the log2FC of healthy + ICS compared to healthy no ICS (Figure 2, Table S5).

### 3.4 Differential Gene Expression Analysis Between Asthma Phenotypes

Previous transcriptomic analyses from airway biopsies and brushes identified mutually exclusive T2 cytokine‐dependent and IL‐17‐dependent gene signatures [7, 8]. In an initial GSVA of 57 bronchial brushings, we observed significant enrichment for the T2 score in T2‐high asthma with a “dose–response” from T2‐high through T2‐intermediate to T2‐low, with a similar, but less significant trend in bronchial biopsies. Conversely, there was a significant increase in IL‐17 score in T2‐low asthma in bronchial brushings, but not biopsies (Figure S5). This further validates the a priori definition of these groups [17].

**FIGURE 3:**
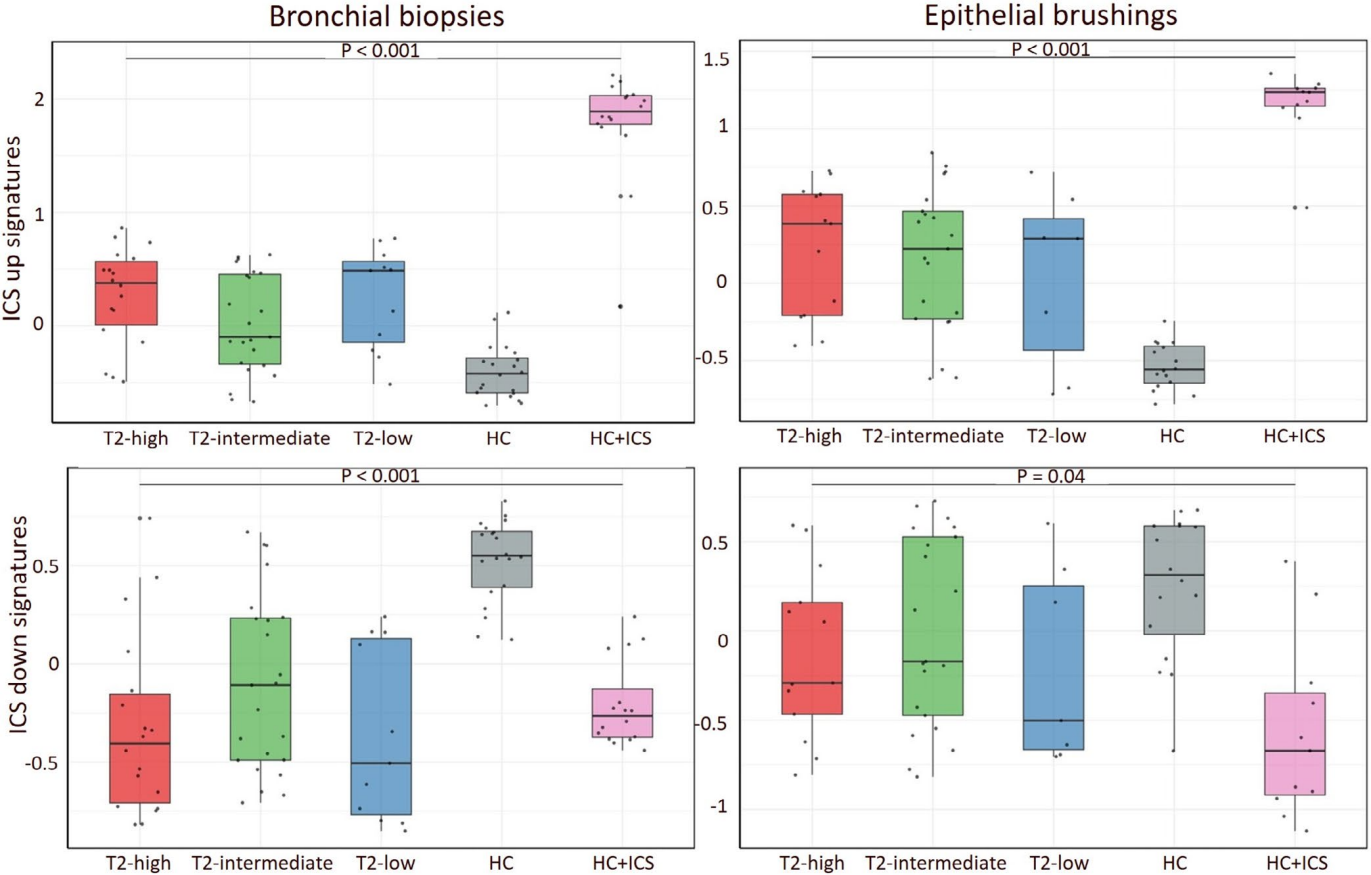
Enrichment score of ICS transactivation and transrepression gene signatures across T2‐high, intermediate, and low asthma, healthy controls, and healthy with ICS with GSVA. ICS transactivation and transrepression gene signatures were generated using the top 20 upregulated and top 20 downregulated genes, respectively, when comparing matched healthy controls pre‐ and post‐treatment with high‐dose ICS for 4 weeks (16).

To assess ICS‐responsiveness in individual patients, we generated 20‐gene transactivation and transrepression signatures using the top 20 upregulated and top 20 downregulated genes common to biopsies and brushes in our previous study investigating the effects of ICS in healthy airways [16]. As expected, there was significant inter‐individual variability in the mean weighted gene expression score, but the group medians were similar across T2‐high, T2‐intermediate, and T2‐low asthma (Figure 3).

### 3.5 Transcriptome Signatures Associated With Key Inflammatory Biomarkers in Asthma

**FIGURE 4:**
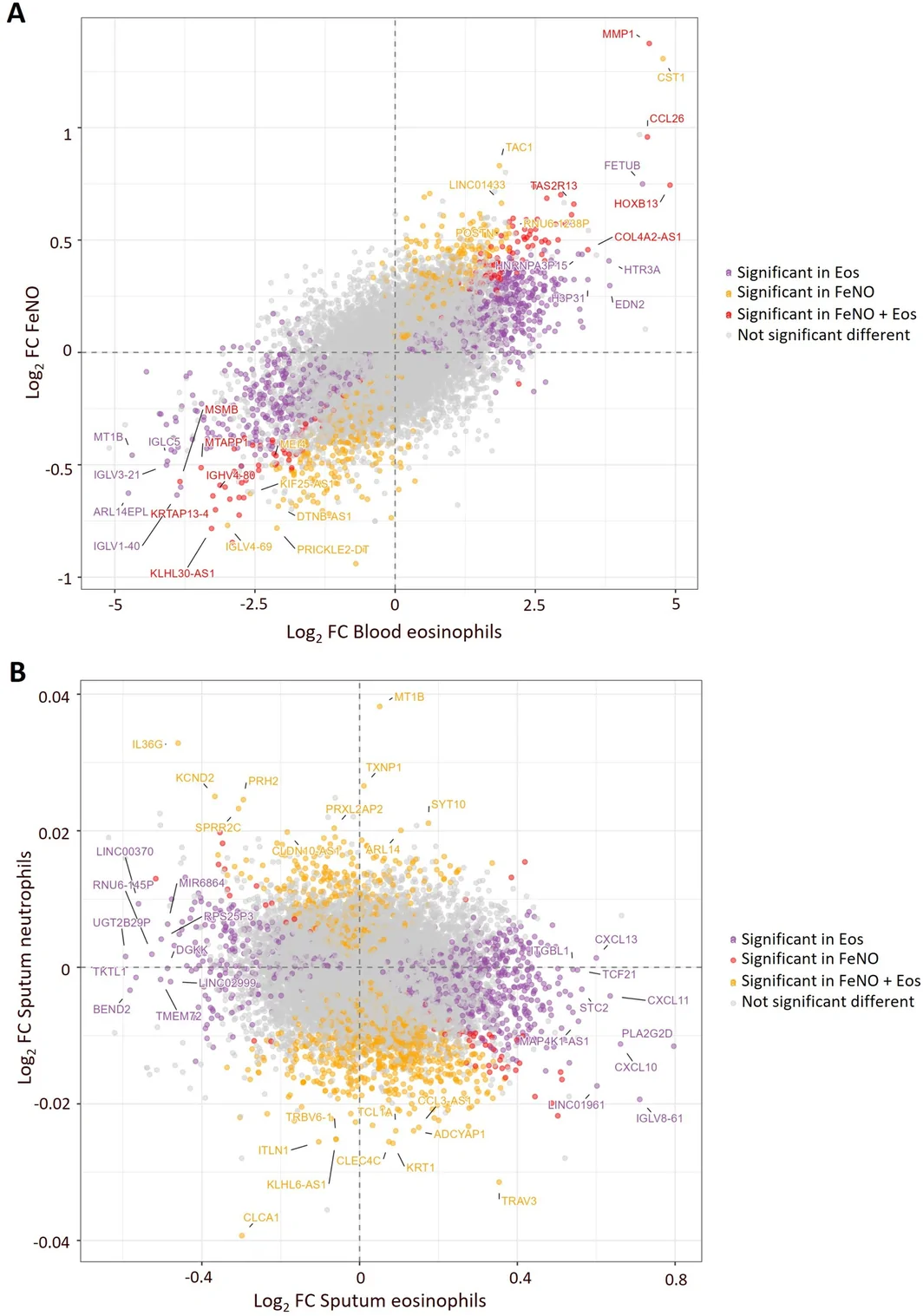
Transcriptomic signatures associated with key inflammatory biomarkers of asthma in bronchial biopsies. (A) Transcriptomic signatures associated with blood eosinophils (x‐axis) and FeNO (y‐axis) in severe asthma. (B) Transcriptomic signatures associated with sputum eosinophils (x‐axis) and sputum neutrophils (y‐axis) in severe asthma. Each point represents a gene plotted by log fold change (logFC) per unit increase in each biomarker. Genes are colour‐coded according to statistical significance: Associated with one biomarker only, both biomarkers, or not significant.

We identified both shared and distinct gene expression patterns of key inflammatory biomarkers in asthma. Many genes were significantly associated with blood eosinophil counts and FeNO, such as *CCL26* and *MMP1*. Some genes were associated only with eosinophils (e.g., *EDN2*), while others showed strong associations with FeNO only (e.g., *POSTN*, *CST1*) (Figure 4A). Distinct gene signatures were linked to sputum eosinophils and sputum neutrophils. Genes like *CXCL10* and *STC2* were associated with eosinophils, whereas *PRH2* and *SYT10* were linked to neutrophils (Figure 4B).

### 3.6 Type‐2 High Asthma

**FIGURE 5:**
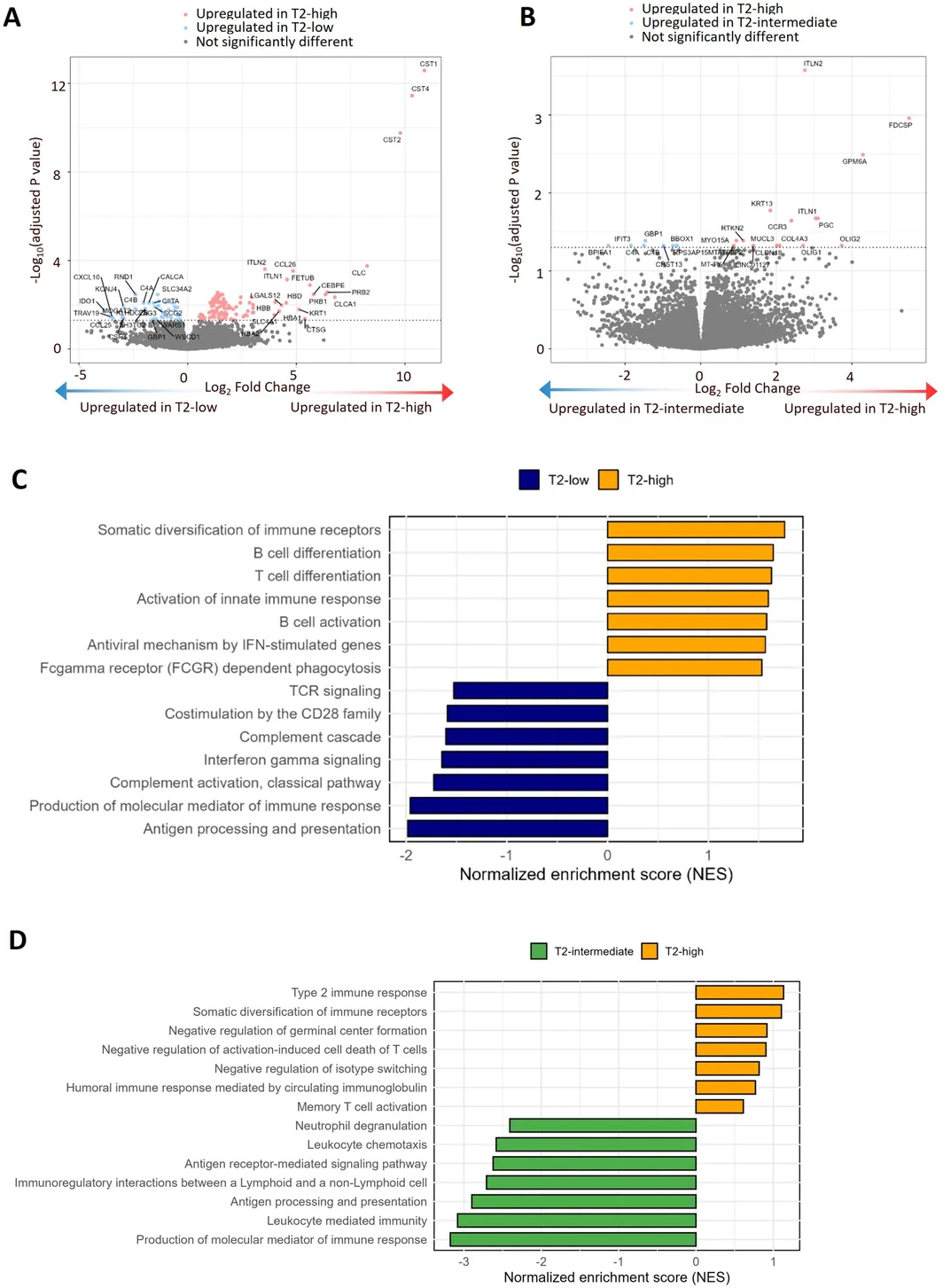
Differential gene expression and immune pathways enrichment in bronchial biopsies from T2‐high, T2‐intermediate, and T2‐low asthma. (A) Volcano plot showing differentially expressed genes between T2‐high and ‐low asthma, adjusted for age, sex, and previous smoking status. Genes upregulated in T2‐high are highlighted in red, and those upregulated in T2‐low are in blue; select genes are annotated. (B) Volcano plot showing differentially expressed genes between T2‐high and ‐intermediate asthma, adjusted for age, sex, and previous smoking status. Genes upregulated in T2‐high are highlighted in red, and those upregulated in T2‐intermediate are in blue; select genes are annotated. (C) Gene set enrichment analysis (GSEA) of immune pathways comparing T2‐high vs. T2‐low asthma. The normalised enrichment score (NES) is shown for selected pathways, with blue bars indicating enrichment in T2‐low and orange bars indicating enrichment in T2‐high. (D) Gene set enrichment analysis (GSEA) of immune pathways comparing T2‐high vs. T2‐intermediate asthma. The normalised enrichment score (NES) is shown for selected pathways, with blue bars indicating enrichment in T2‐intermediate and orange bars indicating enrichment in T2‐high.

Using DESq2, there were 115 genes upregulated and 38 genes downregulated in T2‐high vs. T2‐low asthma in biopsies, but none that were differentially regulated in brushes (Table S6, Figure 5A). T2‐high asthma was characterised by strong upregulation of *CST1/2/4*, *CLC*, and canonical markers of T2 inflammation (*CLCA1*, *FETUB*, *POSTN*, *SERPINB2*, *CCL26*, *CCR3*). In addition, keratin family genes (*KRT1/4*), and putative genes indirectly related to epithelial structure and barrier function (*PRB1/2*, *ROR1*) were upregulated [37], as were *CTSG* (cathepsin G), a secreted neutrophil chymotrypsin, *NTKR2* (neurotrophic tyrosine kinase receptor type 2), implicated in driving cholinergic neuroplasticity in airways disease [38], *CD36* (phosphorylcholine receptor), associated with murine allergic airways disease [39], and *CD44*, which promotes antigen‐specific Th2 cells in murine models [40].

Rank product analysis confirmed these findings but identified a higher number of significant DEGs in both biopsies and brushes when comparing T2‐high and T2‐low asthma, likely reflecting the higher sensitivity and statistical power of the Rank Product method for detecting consistent expression differences across samples (Table S7). Notably, in bronchial biopsies, T2‐high asthma was associated with prominent upregulation of inducible nitric oxide synthase (*NOS2*) and further epithelial barrier and keratin genes (Table S7).

**FIGURE 6:**
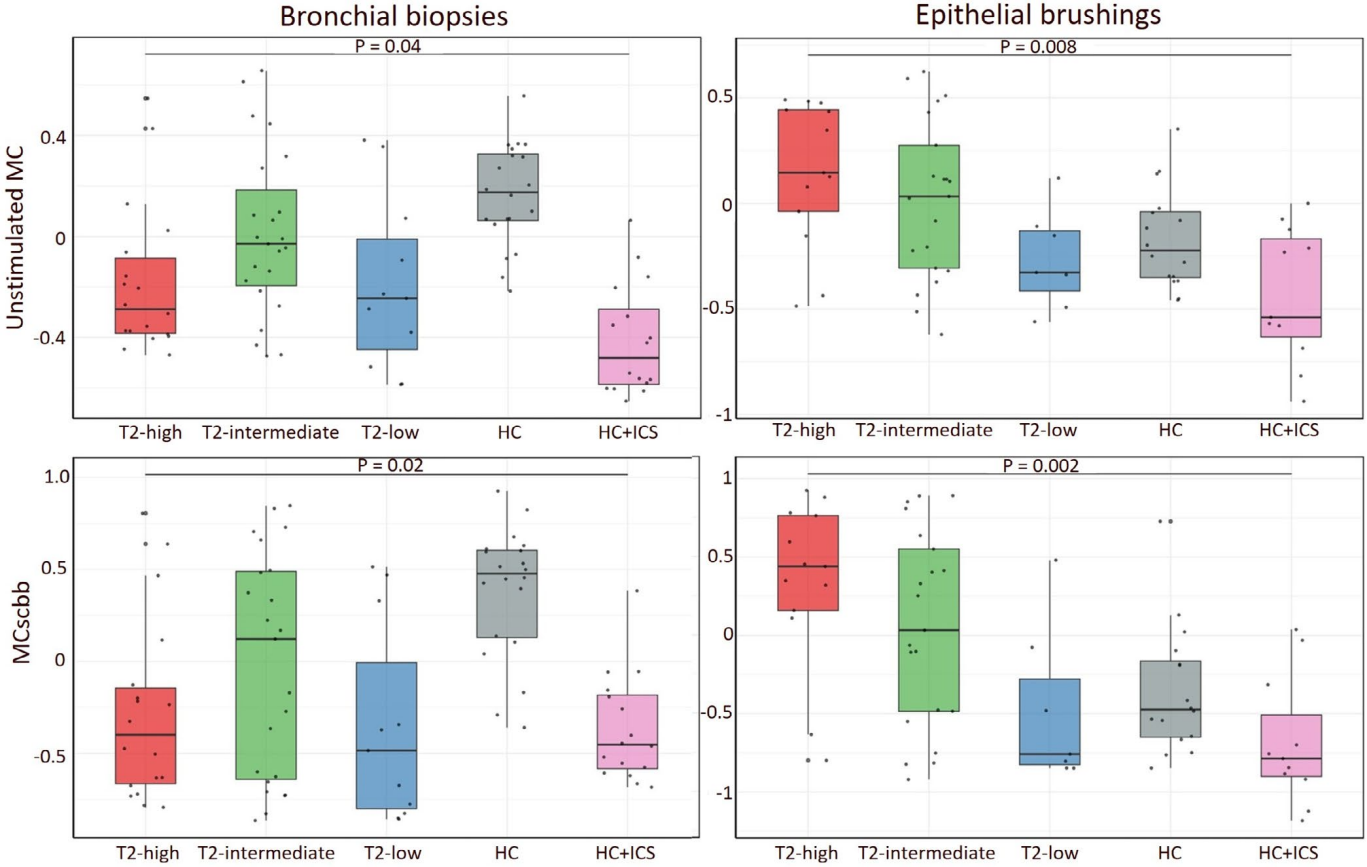
Enrichment score of resting and FcεR1‐activated mast cell gene signatures across T2‐high, intermediate, and low asthma, healthy controls, and healthy with ICS with GSVA. Resting and FcεR1‐activated mast cell gene signatures were generated by Tiotiu et al. (42). Abbreviations: MC: Mast cell; MCscbb: Single‐cell RNA sequencing of MCs from asthma bronchial biopsies.

In bronchial brushes, rank product analysis identified upregulated canonical T2‐dependent genes in T2‐high asthma, and of note, several mast cell‐specific genes (MS4A2, CPA3, tryptases). This was confirmed by analysing mast cell gene signatures described previously [41] (Figure 6).

GSEA of bronchial biopsies showed that T2‐high asthma, when compared with T2‐low or T2 intermediate asthma, was associated with enrichment of pathways for B and T cell adaptive immune responses, increased innate type 1 interferon signalling (Figure 5C,D), and signatures for activated effector CD8 T cells, activated NK cells and CD4 Th17 cells (Table S8). GO terms in T2‐high asthma were related to cell proliferation and histone modifications (Table S9). There was a relative downregulation of biological processes related to ciliary function (Table S9). Deconvolution using the single‐cell atlas in T2‐high asthma predicted an increase in NK cells, lung basal cells, differentiating basal cells, bronchial vessel cells, naïve CD4+ T cells and goblet cells (Table S10).

### 3.7 Type‐2 Low Asthma

Bronchial biopsies in T2‐low asthma demonstrated upregulation of Th1‐ and IL‐17‐associated genes including the chemokines *CXCL9* and *CXCL10*, induced by IFNγ and which activate Th1 cells and are human lung mast cell chemoattractants secreted by ASM, CSF3, a chemokine induced by IL‐17A/F and by IL‐4Rα inhibition [7], and CIITA, a class II transactivator which negatively regulates T cell IL‐4 transcription [42] (Tables S6 and S7 [rank product analysis], Figure 5A). When compared to T2‐high asthma, T2‐low asthma was also associated with higher expression of ASM genes, including the ryanodine receptor (RYR3), the voltage‐gated K^+^ channel *KCNA1* (Kv1.1) and *ACT2A* (α‐smooth muscle actin) (Table S7). There was also marked upregulation of several surfactant proteins, which have anti‐inflammatory and innate immunomodulatory activity.

GSEA of bronchial biopsies demonstrated that T2‐low asthma was associated with enrichment of pathways suggestive of a defence against mucosal pathogens, including antigen processing, TCR activation, CD28 co‐stimulation, IFNγ signalling and complement activation (Figure 5C).

In biopsies, there was relative upregulation of GO biological processes related to ciliary function, with the terms ‘cilium organisation’, ‘cilium movement’ and ‘axoneme assembly’ each among the 5 most enriched processes (Table S9). T2‐low asthma was associated with upregulation specifically of signatures for Treg and for adenosine‐stimulated mast cells (Table S8). Cell deconvolution is summarised in Table S9.

In summary, T2‐low asthma was characterised by the presence of inhibitors of T2‐dependent pathways and upregulation of ASM‐related genes.

### 3.8 Type‐2 Intermediate Asthma

Relatively few genes were upregulated in T2‐intermediate asthma compared with T2‐high asthma (Tables S6, S7; Figure 5B). These included T2‐low‐associated genes such as *GBP1*, complement factors (*C4A, C4B*) in biopsies, and *CXCL10* in brushings. More specific to T2‐intermediate asthma were *IFIT3* (interferon inducible protein) and *BPFIA1* (SPLUNC1), an antimicrobial protein highly secreted in the airways, which inhibits IL‐13‐induced *CCL26* expression.

In bronchial biopsies, T2‐intermediate asthma was associated with enrichment of myeloid leukocyte pathways (Figure 5D), relative upregulation of biological processes related to activated leukocytes, lymphocytes and adaptive T cell responses (Table S11), and upregulation of signatures for leukocyte responses to viruses, including virally activated neutrophils (Table S12). Cell deconvolution showed increases in cells annotated as monocytes, naïve lung CD8 T cells, basophil/mast cells, macrophages and NK cells (Table S13).

### 3.9 Cluster Analyses and Transcription Factor Enrichment Analysis

**FIGURE 7:**
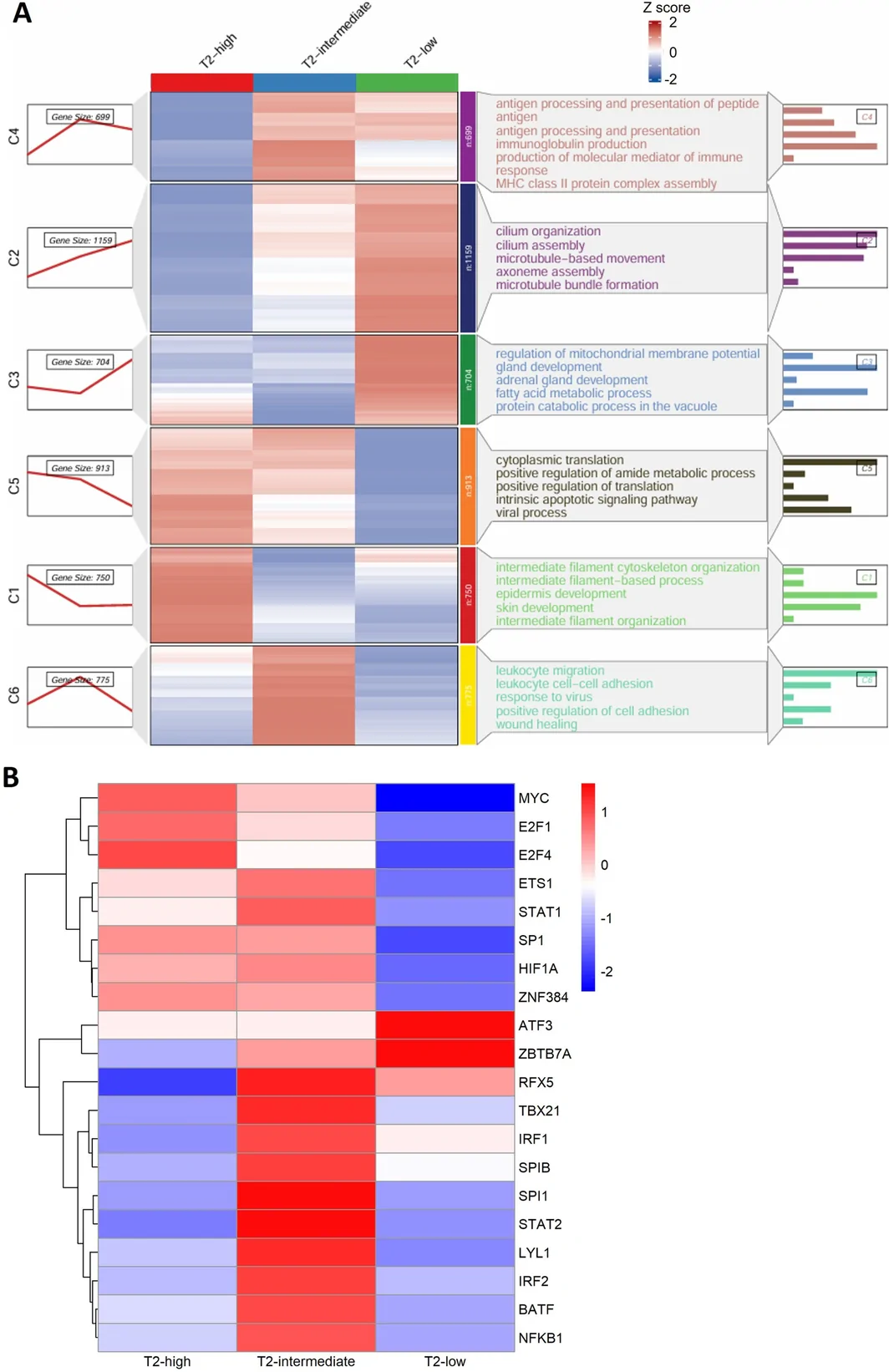
Gene cluster and transcription factor enrichment analysis in bronchial biopsies across T2‐high, intermediate, and ‐low asthma. (A) Gene cluster heatmap showing pathway‐level transcriptomic differences across T2‐high, intermediate, and low asthma in bronchial biopsies. Genes were grouped into distinct co‐expression clusters (C1–C6) using *k*‐means clustering based on shared expression profiles. The line plots on the left show the average expression profile of each gene cluster across the T2 asthma phenotype. The heatmap displays Z‐score‐normalised gene expression values with rows corresponding to genes and columns grouped by T2 asthma phenotype. Red indicates higher expression and blue indicates lower expression. The top enriched Gene Ontology (GO) biological process terms for each cluster are shown on the right. Coloured bars reflect the statistical significance of enrichment. (B) Transcription factor (TF) enrichment across T2‐high, intermediate, and low asthma in bronchial biopsies. The heatmap displays inferred TF activity scores based on the expression of their target genes. Rows represent transcription factors, and columns represent the T2 asthma phenotype. Red indicates higher inferred activity and blue indicates lower activity.

K‐means clustering of bronchial biopsies identified six gene clusters (C1–C6) that distinguished phenotypes (Figure 7A). T2‐high asthma featured upregulation of C1 (epidermal development) and C5 (cytoplasmic translation, viral process), and downregulation of C2 (ciliary organisation), which showed increasing expression from T2‐intermediate to T2‐low asthma and health. T2‐low asthma specifically upregulated C3 (gland development, fatty acid metabolism). T2‐intermediate asthma uniquely showed high expression of C4 (immune mediator, immunoglobulin production) and C6 (leukocyte migration).

The activity of the 20 most differentially expressed transcription factors across phenotypes in bronchial biopsies was inferred (Figure 7B). T2‐high and T2‐intermediate asthma showed common upregulation of eight transcription factors, including *MYC*, *HIF1A*, and *STAT1*, whilst strong upregulation of *ATF3* and *ZBTB7A* was specific to T2‐low asthma. T2‐intermediate asthma was uniquely characterised by specific upregulation of nine factors, including *NFKB1*, *TBX21*, *IRF1*, *IRF2*, *BATF*, and *STAT2*.

WGCNA results are presented in the online supplement, showing that T2‐intermediate asthma was associated positively with the ‘Immune response module’ and ‘Pathogen defence module’. (Figure S6). With WGCNA, T2‐intermediate asthma was positively associated with the immune response, pathogen defence and development and metabolism module (Figure S6).

### 3.10 Validation in the U‐BIOPRED Cohort

Despite U‐BIOPRED having a different patient recruitment strategy and using microarrays rather than RNAseq for gene expression analysis, there were excellent correlations when comparing the log2FC for asthma vs. healthy (no ICS) from RASP and U‐BIOPRED, in both biopsies and brushes for asthma overall (Figure S7), and for the T2‐high vs. T2‐low and T2‐intermediate analyses (Figures S8 and S9). There were relatively few discordant genes. U‐BIOPRED demographic data are presented in Table S14.

## 4 Discussion

For the first time, we have assessed transcriptomic differences between severe asthma and health independent of confounding by therapeutic corticosteroids, as all participants received high‐dose ICS. Many DEGs identified comparing asthma with health are not differentially expressed when comparing asthma to healthy people treated with ICS. Moreover, we have performed stratified analyses of T2‐high, T2‐intermediate, and T2‐low severe asthma, including patients who had undergone protocolised, biomarker‐directed treatment optimisation. The results were validated using the U‐BIOPRED cohort.

When compared with ICS‐treated healthy controls, severe asthma was characterised most by upregulation of mucins (*MU5AC*, *MUC2*) and canonical T2‐related genes. CEACAM5 is a surface receptor on bronchial epithelial cells upregulated in several severe asthma cohorts [31, 43, 44], but downregulated by ICS [16]. It is upregulated by T2 cytokines and may mediate a subset of the IL‐13 transcriptional epithelial signature, most notably modulation of type I interferon signalling [43]. CEACAM5 is also upregulated by IFNγ and common respiratory viruses and used as an adhesion factor by *Haemophilus influenzae* and *Moraxella catarrhalis* [45], so it might contribute to increased bacterial infection in severe asthma [46]. Our data support further investigation of CEACAM5 as a drug target with the CEACAM5‐targeting antibody labetuzumab.

For the T2 biomarkers, FeNO‐high and blood eosinophil‐high patients share many common transcriptome signatures related to T2 inflammation (e.g., *CCL26*) and airways remodelling (e.g., *MMP1*). However, some genes were associated only with eosinophils, such as *EDN2* (endothelin), which may play a role in eosinophil recruitment and activation [47], while some genes were only associated with FeNO, such as *POSTN* and *CST1*, which are linked to IL‐13‐dependent pathways in airway epithelial cells [48]. There was, nevertheless, a strong correlation between blood eosinophils and FeNO. Our study also demonstrated that eosinophilic and neutrophilic inflammation exhibited very different gene expression patterns. Eosinophilic inflammation genes (e.g., *CXCL10, STC2*) and neutrophilic inflammation genes (e.g., *PRH2, SYT10*) were consistent with previous studies [49].

We have previously validated the a priori definition of these T2‐high, intermediate and low groups based on recorded biomarkers and sputum cytokines [17]. The airway T2 and IL‐17‐dependent gene signatures measured in this study further validate the T2 status of these groups. Although these clinical phenotypes appear remarkably similar histologically [17], we have found dysregulated group‐specific inflammatory and remodelling pathways at the molecular level. Comparisons between phenotypes revealed a striking suppression of ciliary gene sets and pathways in T2‐high asthma, which were relatively normal in T2‐intermediate or T2‐low asthma, confirming recent similar observations from U‐BIOPRED [50]. Ciliary dysmotility is described in severe asthma, but the underlying mechanism remains unknown. However, epithelial metaplasia, characterised by replacement of ciliated cells with goblet cells and altered structural (keratins) and adhesion molecule expression (CEACAM5), coupled with excess pathological mucus and altered microbial colonisation, may be contributing factors.

We describe a substantially different range of genes upregulated in T2‐low and intermediate asthma, including CXCL10, a chemokine induced by IFN‐γ, which preferentially attracts activated Th1 cells and mast cells via CXCR3 [51]. In mice, CXCL10 is upregulated by allergen challenge, contributing to AHR, eosinophilia, IL‐4 and CD8 T cell recruitment [52]. In humans, CXCL10 is associated with a mast cell signature and is a marker for exacerbation risk [53], and a predictor of response to omalizumab [54], suggesting an important pathogenic role in T2‐low disease.

Another interesting feature of T2‐low asthma was upregulation of neuroimmune genes, notably SCG2 and CALCA, implicating pathogenic neuroimmune pathways. Secretogranin II (SCG2, chromogranin‐C) is a neuropeptide chemotactic to eosinophils [55]. Pulmonary expression of both SCG2 and CALCA is highly specific to ionocytes. CALCA encodes three peptide hormones: calcitonin, katacalcin and calcitonin gene‐related peptide (CGRP) by tissue‐specific alternative mRNA splicing. In the lungs, aeroallergens induce CGRP release from pulmonary neuroendocrine cells [56], which are in close apposition to CGRP‐receptor expressing ILC2, DCs, basal epithelial cells, and neurons. CGRP can promote Th2 and Th9 responses and has been associated with sensory neuroplasticity and fatal asthma [57].

At a transcriptomic level, the T2‐intermediate phenotype was, perhaps surprisingly, not simply an intermediate stage between T2‐high and T2‐low disease, but was characterised by distinct expression of virus‐activated signatures, i.e., mast cell and NK cell signatures, a pathogen defence gene module and nine distinct transcription factors. The gene most specifically upregulated in this phenotype was BPI Fold Containing Family A Member I (BPFIA1, SPLUNC1). This epithelial protein reduces the surface tension in airway epithelial secretions, promotes adequate mucus clearance and inhibits pathogenic Gram‐negative bacterial biofilms. BPIFA1 inhibits IL‐13‐mediated eotaxin‐3 and ASM contraction. However, it is markedly induced by exposure to epoxy chemicals in sensitised individuals, and by secretoglobin (SCGB1A1, CC16), recruiting macrophages and neutrophils, implying a proinflammatory role [58].

Clinically, people with severe asthma are insensitive to the therapeutic effects of ICS. Our data show that at the molecular level, this corticosteroid insensitivity is highly heterogeneous. The T2 inflammatory pathway is clearly insensitive to ICS in T2 biomarker‐high patients. However, the majority of genes which demonstrate transactivation or transrepression by ICS in healthy airways show concordant changes in asthma compared to health (Figure 2), indicating that many genes and their related pathways remain corticosteroid‐sensitive. As the majority of people with T2 low‐severe asthma have been eosinophilic in the past, it is likely that T2 activity in their airways is sensitive to ICS and therefore suppressed. As suggested previously [17], and supported by the data here, it is likely that residual disease expression in T2 low asthma is driven by ASM dysfunction, non‐T2‐driven mucus expression, and mast cell mediators. The mechanism of selective corticosteroid resistance in key pathways such as the T2 pathway remains a major unanswered question in asthma.

Our study has limitations as it was cross‐sectional, with bronchoscopies performed only at a stable state and not during exacerbations. Furthermore, as bulk RNAseq was used, we could not measure individual cell types directly or assess cell‐type‐specific gene expression or spatial information. In addition, patients meeting the T2‐low definition were relatively difficult to find in our severe asthma clinics, and so the number recruited was relatively small. Yet despite this, we have identified highly significant molecular features associated with this group.

In summary, we have characterised three major severe asthma phenotypes, independent of confounding by therapeutic corticosteroids, identifying gene sets and pathways distinct to each. Corticosteroid‐resistant T2‐high severe asthma was characterised by upregulated T2‐high genes, epithelial mast cell genes, epithelial barrier genes, and keratins; T2‐low severe asthma was characterised by Th1‐ and IL‐17‐associated genes, neuroimmune genes, and ASM genes; steroid‐responsive T2‐intermediate asthma was characterised by signatures of pathogen defence, virus activation, enrichment for mast and NK cells, and six distinct transcription factors. These insights should guide future efforts to identify novel treatment targets for these less well‐studied phenotypes.

Further discussion points are covered in the online supplement.

## Author Contributions

**J.S.:** performed transcriptomic data analysis. **R.C.:** principal investigator in Glasgow. **S.B.:** performed bronchoscopy in Glasgow. **A.H.M.:** principal investigator in Birmingham. **R.S.:** recruited and bronchoscoped patients in Oxford. **I.D.P.:** principal investigator in Oxford. **S.J.F.:** principal investigator in Manchester, performed bronchoscopy. **V.B.:** sample management and storage. **L.P.M.:** co‐Investigator in Belfast performed bronchoscopies in Belfast, on the study progress committee. **P.H.H.:** principal investigator in Southampton. **S.‐E.D., I.M.A. and N.Z.K.:** contributed U‐BIOPRED data and interpretation on behalf of the U‐BIOPRED consortium. **J.R.A.:** contributed to study design. **L.G.H.:** principal investigator in Belfast, conceived and designed the study, academic lead for RASP‐UK programme, bronchoscopy study progress committee. **D.C.:** contributed to study design, supervised RNAseq experiments, and contributed to transcriptomic analysis. **T.S.C.H.:** contributed to transcriptomic data analysis and interpretation, and drafted the manuscript. **E.M.:** performed transcriptomic data analysis. **P.B.:** conceived and designed the study, wrote the study protocol and ethical application, analysed the data, drafted the manuscript, and performed all bronchoscopies in Leicester. Chief investigator. All authors reviewed the data and contributed to its interpretation, edited the manuscript, and approved the final submitted version.

## Funding

This paper was supported by the Medical Research Council (MR/M016579/1), and in part by the National Institute for Health and Care Research (NIHR) Leicester Biomedical Research Centre (Respiratory) and the NIHR Oxford Biomedical Research Centre. Additional funding was received from Genentech to the University Hospitals of Leicester NHS Trust to support this study. SJF is supported by the NIHR Manchester Biomedical Research Centre. TSCH was supported by the Wellcome Trust (211050/Z/18/z). The views expressed are those of the author(s) and not necessarily those of the NHS, the NIHR or the Department of Health. For the purpose of Open Access, the author has applied a CC BY public copyright licence to any Author Accepted Manuscript version arising from this submission.

## Conflicts of Interest

R.C. has received lecture fees from GSK, AstraZeneca, Chiesi, and Sanofi; honoraria for Advisory Board Meetings from GSK, AstraZeneca, and Celltrion; Consultant to Connect Biopharma; sponsorship to attend international scientific meetings from Sanofi and a research grant from AstraZeneca for a UK multi‐centre study. A.H.M. has received personal and institutional payment for talks, advisory board meetings, education and research funding from GSK, AstraZeneca, Teva, Chiesi, NAPP, Sanofi, Novartis, PI. RS received payment or honoraria from AstraZeneca and GSK for lectures, presentations, speakers' bureaus, manuscript writing or educational events. He also received funding from AstraZeneca and Chiesi to support attending meetings and/or travel. I.D.P—In the last 5 years, I.D.P. has received speaker's honoraria for speaking at sponsored meetings from AstraZeneca, Boehringer Ingelheim, Aerocrine, Almirall, Novartis, Teva, Chiesi, Sanofi/Regeneron, Menarini and GSK, and payments for organising educational events from AstraZeneca, GSK, Sanofi/Regeneron and Teva. He has received honoraria for attending advisory panels with Genentech, Sanofi/Regeneron, AstraZeneca, Boehringer Ingelheim, GSK, Novartis, Teva, Merck, Circassia, Chiesi and Knopp, and payments to support FDA approval meetings from GSK. He has received sponsorship to attend international scientific meetings from Boehringer Ingelheim, GSK, AstraZeneca, Teva and Chiesi. He has received a grant from Chiesi to support a phase 2 clinical trial in Oxford. In 2014–5 and 2019–20, he was an expert witness for a patent dispute involving AstraZeneca and Teva. L.P.M. declares Research funding from Chiesi and Merck; consultancies for Chiesi, Glaxo Smith Kline, Merck, Sanofi, Genentech and support to attend scientific meetings from Chiesi, Merck and Bayer. P.H.H. is an employee of GSK. SED declares research funding from the Swedish Heart Lung Foundation, The Stockholm County Council Clinical Research Funds (ALF), AstraZeneca, and GSK; consultancies for AstraZeneca, GSK, and Sanofi; Joint Steering Committee membership for 3TR (IMI project). I.M.A. reports institutional grants from MRC, EPSRC, EU, GSK, Sanofi, and Sysmex; consulting fees from GSK; lecture and travel expenses from AstraZeneca, GSK, Sanofi, and Sunvou; serving on advisory boards for Kinaset and GSK; and is the head of the asthma section for EAACI. J.R.A. was an employee of Genentech. L.G.H. reports grants from Genentech/Hoffman la Roche, during the conduct of the study; other from AstraZeneca, Boehringer Ingelheim, Chiesi, GSK and Napp Pharmaceuticals, personal fees from Novartis, Hoffman la Roche/Genentech Inc., Sanofi, Evelo Biosciences, Glaxo Smith Kline, AstraZeneca, Teva, Theravance, Circassia, grants from Medimmune, Novartis UK, Roche/Genentech Inc., and Glaxo Smith Kline, Amgen, Genentech Hoffman la Roche, AstraZeneca, Medimmune, Glaxo Smith Kline, Aerocrine and Vitalograph, outside the submitted work. DC is an employee of Genentech. T.S.C.H. has received grants from Pfizer Inc., grants from the University of Oxford, grants from the Wellcome Trust, grants from the Medical Research Council, grants from The Guardians of the Beit Fellowship, and grants from the NIHR Oxford Biomedical Research Centre during the conduct of the study; and personal fees from Amgen, AstraZeneca/Pieris, Chiesi and Sanofi outside the submitted work. P.B. has received research funding from Genentech via the University Hospitals of Leicester NHS Trust; DEShaw Research via the University of Leicester. Consultancies for Genentech and DEShaw Research via the University of Leicester. One honorarium from AstraZeneca. Support to attend scientific meetings from GSK and Sanofi Genzyme. The other author declare no conflicts of interest.

## Supporting information


**Figure S1:** Summary of study design.
**Figure S2:** Correlations between clinical characteristics in asthma. (A) Correlation matrix of clinical variables. Spearman correlation matrix showing pairwise associations between clinical characteristics. Circle colour indicates the direction of correlation (red = positive, blue = negative), and circle size reflects the correlation strength. Stars indicate statistical significance of the correlation: p < 0.05: *, p < 0.01: **, p < 0.001: ***. (B) Scatter plot of the correlation between fractional exhaled nitric oxide (FeNO) levels and blood eosinophil counts (cells × 10^9/L) in patients with asthma. Both axes are logarithmic. A linear regression line (red) with a 95% confidence interval (grey) is fitted to the data. (Spearman's R = 0.45, p = 0.013), indicating a moderate, positive correlation between FeNO levels and blood eosinophil counts in this patient population.
**Figure S3:** Heatmap of differentially expressed genes across asthma, healthy with ICS, and healthy control groups in bronchial biopsies. The heatmap displays the expression levels of selected asthma signature genes across three groups: Asthma patients (orange), healthy controls using inhaled corticosteroids (ICS; pink), and healthy controls not using ICS (grey). Each row represents one gene, and each column represents one individual sample. Gene expression values are z‐score normalised across samples, with red indicating higher expression and blue indicating lower expression. Genes were selected based on established asthma signatures from Table 2.
**Figure S4:** Cell type composition of asthma and healthy controls. (A) Deconvolution analysis based on the Travaglini Lung Atlas comparing asthma vs. healthy controls not receiving ICS. (B) Deconvolution analysis based on the Travaglini Lung Atlas (B) comparing different phenotypes of asthma. Boxplots indicate median and interquartile ranges. Statistical significance was assessed using ANOVA.
**Figure S5:** Type 2 (T2) and IL‐17 signature expression across T2‐high, ‐intermediate, and ‐low asthma, healthy controls. (A) T2 and IL‐17 signature scores used IL‐13–induced genes (POSTN, SERPINB2, and CLCA1) and IL‐17–induced chemokines (CXCL1, CXCL2, CXCL3, IL8, and CSF3) to estimate Type 2 (T2) and IL‐17 immune activity. Healthy controls were not receiving ICS. Signature scores were derived by averaging log2‐transformed signature gene expression counts that have been normalised by sample library size, plus 1 (to accommodate genes with a normalised count of 0). (B) The correlation between T2 and IL‐17 signature scores in asthma.
**Figure S6:** Weighted gene co‐expression network analysis (WGCNA) on bronchial biopsy differentially expressed genes in asthma. (A) Clustering of module eigengenes based on their correlation. Modules with similar expression profiles across samples are grouped together, indicating higher‐order relationships among co‐expression modules. (B) Clustering of module eigengenes based on their correlation. Modules with similar expression profiles across samples are grouped together, indicating higher‐order relationships among co‐expression modules. (C) Gene clustering dendrogram based on topological overlap, with assigned module colours displayed below. Each branch corresponds to a gene cluster (module) identified by dynamic tree cutting, with colour assignments representing distinct modules. (D) Modules were named according to their top significantly enriched Gene Ontology (GO) biological process term. Each cell displays the correlation coefficient (top) and the corresponding *p*‐value (bottom, in parentheses). Blue indicates negative correlations, and red indicates positive correlations; more saturated colours represent stronger associations. (E) Gene Ontology (GO) enrichment analysis of selected WGCNA modules. The dot plot shows representative GO biological process terms enriched in the selected modules. Dot size reflects the proportion of genes in each module associated with the GO term (GeneRatio), and dot colour represents the adjusted *p*‐value. ME, module eigengene. For sex, we compare female vs. male.
**Figure S7:** Log2 fold change of asthma vs. health (no ICS) in RASP plotted against asthma vs. health no ICS in U‐BIOPRED. (A) Biopsies; (B) Brushes.
**Figure S8:** Log2 fold change of T2‐high vs. T2‐low asthma in RASP plotted against T2‐high vs. T2‐low asthma in U‐BIOPRED. (A) Biopsies; (B) Brushes.
**Figure S9:** Log2 fold change of T2‐high vs. T2‐intermediate asthma in RASP plotted against T2‐high vs. T2‐low asthma in U‐BIOPRED. (A) Biopsies; (B) Brushes.
**Table S1:** All differentially expressed genes between asthma vs. health in bronchial biopsies and brushings. All differentially expressed genes between asthma vs. healthy controls receiving ICS; asthma vs. healthy controls not receiving ICS.
**Table S2:** Gene Ontology Biological Process pathways enriched in asthma vs. healthy controls receiving ICS in bronchial biopsies. Gene Ontology (GO) Biological Process pathways significantly enriched between asthma patients and healthy controls receiving inhaled corticosteroids were identified using rank product analysis. Pathways shown are ordered by *p* value. The x‐axis *in the* figure represents approximate gene ranks. NES, normalised enrichment score.
**Table S3:** Immune response pathways enriched in asthma vs. healthy controls receiving ICS in bronchial biopsies. Immune response pathways significantly enriched between asthma patients and healthy controls receiving inhaled corticosteroids were identified using rank product analysis. Pathways shown are ordered by *p* value. The x‐axis *in the* figure represents approximate gene ranks. NES, normalised enrichment score.
**Table S4:** Lung cell type pathways enriched in asthma vs. healthy controls receiving ICS in bronchial biopsies.
Lung cell type pathways significantly enriched between asthma patients and healthy controls receiving inhaled corticosteroids were identified using rank product analysis. Pathways shown are ordered by *p* value. The x‐axis in the figure represents approximate gene ranks. NES, normalised enrichment score.
**Table S5:** The log2 fold change for asthma vs. health, and healthy vs. healthy + ICS, for all detected genes in asthma. This was used to generate Figure 2.
**Table S6:** All differentially expressed genes between T2‐high asthma vs. other phenotypes in bronchial biopsies and brushings. All differentially expressed genes between T2‐high asthma vs. T2‐low asthma, between T2‐high asthma vs. T2‐intermediate asthma, in biopsies and in brushings. Differential expression analysis was performed using the *DESeq2* package.
**Table S7:** Rank product analysis of differentially expressed genes between T2‐high asthma vs. other phenotypes in bronchial biopsies and brushings. All differentially expressed genes between T2‐high asthma vs. T2‐low asthma, between T2‐high asthma vs. T2‐intermediate asthma, in biopsies and in brushings. Differential expression was assessed using the non‐parametric, rank‐based method implemented in the *RankProd* package.
**Table S8:** Immune response pathways enriched between T2‐high and T2‐low asthma in bronchial biopsies. Immune response pathways significantly enriched between T2‐high and T2‐low asthma were identified using rank product analysis. Pathways shown are ordered by *p* value. The x‐axis in the figure represents approximate gene ranks. NES, normalised enrichment score.
**Table S9:** Gene Ontology Biological Process pathways enriched between T2‐high and T2‐low asthma in bronchial biopsies. Gene Ontology Biological Process pathways significantly enriched between T2‐high and T2‐low asthma were identified using rank product analysis. Pathways shown are ordered by *p* value. The x‐axis in the figure represents approximate gene ranks. NES, normalised enrichment score.
**Table S10:** Lung cell type pathways enriched between T2‐high and T2‐low asthma in bronchial biopsies.
Lung cell type pathways significantly enriched between T2‐high and T2‐low asthma were identified using rank product analysis. Pathways shown are ordered by *p* value. The x‐axis in the figure represents approximate gene ranks. NES, normalised enrichment score.
**Table S11:** Gene Ontology Biological Process pathways between T2‐high and T2‐intermediate asthma in bronchial biopsies. Gene Ontology Biological Process pathways significantly enriched between T2‐high and T2‐intermediate asthma were identified using rank product analysis. Pathways shown are ordered by *p* value. The x‐axis in the figure represents approximate gene ranks. NES, normalised enrichment score.
**Table S12:** Immune response pathways enriched between T2‐high and T2‐intermediate asthma in bronchial biopsies. Immune response pathways significantly enriched between T2‐high and T2‐low asthma were identified using rank product analysis. Pathways shown are ordered by *p* value. The x‐axis in the figure represents approximate gene ranks. NES, normalised enrichment score.
**Table S13:** Lung cell type pathways enriched between T2‐high and T2‐intermediate asthma in bronchial biopsies. Lung cell type pathways significantly enriched between T2‐high and T2‐intermediate asthma were identified using rank product analysis. Pathways shown are ordered by *p* value. The x‐axis in the figure represents approximate gene ranks. NES, normalised enrichment score.
**Table S14:** Clinical characteristics of participants in the U‐BIOPRED cohort.

## Data Availability

The data that support the findings of this study are openly available in Gene Expression Omnibus (GEO) at https://www.ncbi.nlm.nih.gov/geo/query/acc.cgi?acc=GSE242048, reference number GSE242048 and GSE301357.
