# Supplementary material for: The Airway Transcriptome in Type 2 Cytokine Biomarker‐High and ‐Low Severe Asthma
Source: Allergy. 2026 Jul 20;81(9):3300–17. doi: 10.1111/all.70441 (PMC13569630; doi:10.1111/all.70441)
Supplement: Supplementary file 1 [file ALL-81-3300-s001.zip › all70441-sup-0001-Supinfo/all70441-sup-0002-Supinfo.docx]

**ONLINE SUPPLEMENT**

**The airway transcriptome in type 2 cytokine biomarker-high and -low severe asthma**

^1^Jiashu Shen PhD, ^2^Rekha Chaudhuri MD, ^2^Stephen Bicknell FRCP, ^3^Adel H. Mansur FRCP, PhD, ^1^Rahul Shrimanker MD, PhD, ^1^Ian D. Pavord FMedSci, ^4^Stephen J. Fowler FRCP, MD, ^5^Vanessa Brown, PhD, ^5^Lorcan P. McGarvey FRCP, MD, ^6^Peter H. Howarth FRCP, DM, ^7^Sven-Erik Dahlén^#^, ^8^Ian M. Adcock, ^8^Nazanin Zounemat-Kermani^#^,^9^Joseph R. Arron MD, PhD, ^5^Liam G. Heaney MRCP, MD, ^9^David F. Choy BSc, ^1^Timothy S.C. Hinks MRCP, PhD*, ^1^Emanuele Marchi PhD*, ^10^Peter Bradding FRCP, DM*; on behalf of the UK Medical Research Council Refractory Asthma Stratification programme (RASP-UK)

*These authors contributed equally

^#^ On behalf of the U-BIOPRED Consortia

^1^NIHR Oxford Respiratory BRC, Nuffield Department of Medicine, University of Oxford, Oxford, UK**.**

^2^Gartnavel General Hospital, Glasgow, and Institute of Infection& Immunity, University of Glasgow, Glasgow, UK.

^3^University of Birmingham and Heartlands Hospital, University Hospitals Birmingham NHS Foundation Trust, Birmingham, UK.

^4^School of Biological Sciences, Faculty of Biology, Medicine and Health, University of Manchester, Manchester Academic Health Science Centre and NIHR Manchester Biomedical Research Centre, Manchester University Hospitals NHS Foundation Trust, Manchester, UK.

^5^ Wellcome-Wolfson- Centre for Experimental Medicine, Queen's University Belfast School of Medicine Dentistry and Biomedical Sciences, Belfast, UK.

^6^School of Clinical and Experimental Sciences, University of Southampton, NIHR Southampton Biomedical Research Centre, Southampton, UK

^7^Department of Medicine Huddinge, and, Institute of Environmental Medicine, Karolinska Institutet, Stockholm, Sweden; Centre for Allergy Research, Karolinska Institutet, Stockholm, Sweden & Department of Respiratory Medicine and Allergy, Karolinska University Hospital, Stockholm, Sweden

^8^National Heart and Lung Institute, Imperial College, London, UK & Data Science Institute, Imperial College London, London, UK.

^9^Genentech, Inc. South San Francisco, CA, USA.

^10^ Department of Respiratory Sciences, University of Leicester, Leicester Respiratory NIHR BRC, Glenfield Hospital, Leicester, UK.

**Corresponding author**

Professor Peter Bradding, Department of Respiratory Sciences, University of Leicester, Glenfield Hospital, Leicester, LE3 9QP, UK. Tel: +44 116 258 3998

E-mail: [pb46@le.ac.uk](mailto:pb46@le.ac.uk)

**Running title:** The airway transcriptome of T2-high and T2-low asthma

Contents

[**SUPPLEMENTARY METHODS**	5](#_Toc219816729)

[Study population	5](#_Toc219816730)

[Inclusion criteria for people with severe asthma in RASP	7](#_Toc219816731)

[Inclusion criteria for healthy volunteers in the Leicester healthy volunteer study (HVS)	11](#_Toc219816732)

[RNA sequencing	12](#_Toc219816733)

[Gene expression analysis	13](#_Toc219816734)

[Statistical analysis	14](#_Toc219816735)

[Validation using the U-BIOPRED bronchoscopy cohort	15](#_Toc219816736)

[**SUPPLEMENTARY RESULTS**	17](#_Toc219816737)

[T2-high versus T2-low asthma	17](#_Toc219816738)

[T2-low asthma	18](#_Toc219816739)

[T2-intermediate asthma	19](#_Toc219816740)

[**SUPPLEMENTARY DISCUSSION**	21](#_Toc219816741)

[**SUPPLEMENTARY REFERENCES**	26](#_Toc219816742)

[**SUPPLEMENTARY TABLES**	29](#_Toc219816743)

[**Supplementary Table S1.** All differentially expressed genes between asthma versus health in bronchial biopsies and brushings.	29](#_Toc219816744)

[**Supplementary Table S2.** Gene Ontology Biological Process pathways enriched in asthma *versus* healthy controls receiving ICS in bronchial biopsies.	30](#_Toc219816745)

[**Supplementary Table S3**. Immune response pathways enriched in asthma *versus* healthy controls receiving ICS in bronchial biopsies.	31](#_Toc219816746)

[**Supplementary Table S4**. Lung cell type pathways enriched in asthma *versus* healthy controls receiving ICS in bronchial biopsies.	32](#_Toc219816747)

[**Supplementary Table S5.** The log2 fold change for asthma versus health, and healthy versus healthy + ICS, for all detected genes in asthma. This was used to generate figure 2	33](#_Toc219816748)

**Supplementary** [**Table S6**. All differentially expressed genes between T2-high asthma *versus* other phenotypes in bronchial biopsies and brushings.	34](#_Toc219816749)

[**Supplementary Table S7**. Rank product analysis of differentially expressed genes between T2-high asthma *versus* other phenotypes in bronchial biopsies and brushings.	35](#_Toc219816750)

[**Supplementary Table S8.** Immune response pathways enriched between T2-high and T2-low asthma in bronchial biopsies.	36](#_Toc219816751)

[**Supplementary Table S9.** Gene Ontology Biological Process pathways enriched between T2-high and T2-low asthma in bronchial biopsies.	37](#_Toc219816752)

[**Supplementary Table S10**. Lung cell type pathways enriched between T2-high and T2-low astma in bronchial biopsies.	38](#_Toc219816753)

[**Supplementary Table S11.** Gene Ontology Biological Process pathways between T2-high and T2-intermediate asthma in bronchial biopsies.	3](#_Toc219816754)9

[**Supplementary Table S12.** Immune response pathways enriched between T2-high and T2-intermediate asthma in bronchial biopsies.	40](#_Toc219816755)

[**Supplementary Table S13.** Lung cell type pathways enriched between T2-high and T2-intermediate asthma in bronchial biopsies.	41](#_Toc219816756)

[**Supplementary table S14.** Clinical characteristics of participants in the U-BIOPRED cohort.	42](#_Toc219816757)

[**SUPPLEMENTARY FIGURES**	44](#_Toc219816758)

[**Figure S1.** Summary of study design	45](#_Toc219816759)

[**Figure S2.** Correlations between clinical characteristics in asthma.	46](#_Toc219816760)

[**Figure S3.** Heatmap of differentially expressed genes across asthma, healthy with ICS, and healthy control groups in bronchial biopsies.	48](#_Toc219816761)

[**Figure S4.** Cell type composition of asthma and healthy controls	49](#_Toc219816762)

[**Figure S5.** Type 2 (T2) and IL-17 signature expression across T2-high, -intermediate, and -low asthma, healthy controls.	50](#_Toc219816763)

[**Figure S6.** Weighted gene co-expression network analysis (WGCNA) on bronchial biopsy differentially expressed genes in asthma.	51](#_Toc219816766)

[**Figure S7.** Log2 fold change of asthma versus health (no ICS) in RASP plotted against asthma versus health no ICS in U-BIOPRED.	53](#_Toc219816767)

[**Figure S8.** Log2 fold change of T2-high versus T2-low asthma in RASP plotted against T2-high versus T2-low asthma in U-BIOPRED.	54](#_Toc219816768)

[**Figure S9.** Log2 fold change of T2-high versus T2-intermediate asthma in RASP plotted against T2-high versus T2-low asthma in U-BIOPRED.	55](#_Toc219816769)

# SUPPLEMENTARY METHODS

**Ethics and consent**

The United Kingdom Refractory Asthma Stratification Programme (RASP) bronchoscopy study was a prospective multi-centre study, which recruited people with severe asthma prospectively using pre-defined inclusion and exclusion criteria. It was approved by the East Midlands - Leicester South Research Ethics Committee (REC) (reference 16/EM/0260) and registered at ClinicalTrials.gov (NCT02883530)(1). Biopsy and brush samples collected using the same standard operating procedure were also used from the pre- and post-intervention arms of a second, single centre bronchoscopy study assessing the effects of ICS on adult healthy volunteers (referred to as Leicester HVS from here) (NCT02476825, REC approval 15/EM/0313)(2). All participants gave written informed consent. The Leicester HVS ran contemporaneously with RASP and used the same standard operating procedure and the same bronchoscopist for both studies (PB).

## Study population

Detailed inclusion and exclusion criteria are provided below. People with asthma aged 18-70 were eligible, and all were current non-smokers with a <15 pack-year smoking history.

Standard criteria for the diagnosis of asthma were used. Patients were deemed adherent in their asthma centre through the prior analysis of prescription refills, measurement of prednisolone and cortisol levels if appropriate, and/or FeNO suppression testing(3, 4). Current use of a biologic treatment was an exclusion criteria, and only one patient had used a biologic previously (omalizumab).

Participants underwent extensive evaluation at baseline including a full medical history, lung function testing, bronchial challenge using methacholine where appropriate, and induced sputum.

People with severe asthma were recruited prospectively and identified as follows: i) patients with a previous FeNO ≥45 ppb and blood eosinophils ≥0.3x10^9^/L, who did not suppress their FeNO during a FeNO suppression test(3-5) during routine clinical care, referred to as T2 high; ii) patients with a FeNO ≤30 ppb and blood eosinophils ≤0.2x10^9^/L identified in clinic, referred to as T2 biomarker-low (T2-low); iii) patients who had exited the RASP T2-biomarker (FeNO, blood eosinophils, periostin)-driven treatment optimisation study(6) with intermediate biomarker measurements, referred to as T2-intermediate or T2-low (as described for ii above).

The rationale for the FeNO and blood eosinophil values defining group the T2-low group was based on analysis of our existing cohorts of healthy subjects and patients with severe asthma in 2015 when the study was designed. The FeNO upper 95% CI of the mean was 29 ppb in our previous cohorts of healthy subjects, while the American Thoracic Society consider <25 ppb normal. The upper 95% CI of the geometric mean for blood eosinophils in our cohorts of healthy subjects was 0.16x10^9^/L, while the lower 95% CI for blood eosinophils in patients with a T2-dependent gene signature was 0.31 x10^9^/L, and the lower 95% CI for blood eosinophils for those with an IL-17 signature was 0.27 x10^9^/L. Based on these cut values and our prior definition of a T2-high gene signature, we would be unlikely to identify T2-high patients based on the proposed cut-offs in the T2-low group (T2 and IL-17 gene signature data from reference (7)).

Healthy volunteers in Leicester HVS had no prior history or clinical evidence of lower respiratory disease, and normal spirometry. Healthy volunteers with a history of rhinitis (perennial or seasonal) were required to have a PC_20_ methacholine >16 mg/ml. Bronchoscopies were performed before and after participants received inhaled fluticasone propionate 500 mcg bd daily for 4 weeks. This study has been published previously (2).

The study is summarised in **Figure S1**.

**Patient populations**

## Inclusion criteria for people with severe asthma in RASP

People with asthma met the following criteria for study entry:

1.	Ability and willingness to provide written informed consent and to comply with the study protocol

2.	Age 18–70 years at the time of informed consent

3.	Severe asthma (BTS treatment step 4/5) despite intensive follow-up by an asthma specialist for at least 3 months

4.	Diagnosis of asthma at least 12 months prior to informed consent

5.	Baseline post bronchodilator FEV1 ≥ 40% of predicted

6.	History of asthma treatment with high doses of inhaled glucocorticosteroids (≥ 1000 μg fluticasone propionate daily or equivalent (Clenil [BDP] 2000 μg, Fostair [BDP] 800 μg, fluticasone furoate 192 μg, budesonide dry powder 1600 μg, ciclesonide 640 μg), and LABA, with or without an additional controller, for at least 3 months prior to screening, or prior to corticosteroid optimisation as part of the RASP biomarker-guided study (6).

7.	For patients using oral corticosteroids, adherence with their oral prednisolone regimen, as demonstrated by detectable serum prednisolone and evidence of suppressed cortisol within 6 hours of reported daily dose, on at least one occasion during the screening period or within the last 12 months is required.

8.	Assessment according to the standards of the BTS UK Difficult Asthma Network or equivalent

9.	Chest X-ray or computed tomography (CT) scan obtained within 12 months prior to consent (Visit 1) or chest X-ray during the screening period (prior to Visit 2) confirming the absence of other clinically significant lung disease

10.	Documented history of bronchodilator reversibility response of ≥ 12% and ≥ 200 mL within the past 24 months, as demonstrated by any of the following:

i)	Documented airflow obstruction (FEV_1_/forced vital capacity [FVC] <70%), where FEV_1_ has varied by ≥ 12% either spontaneously or in response to oral corticosteroid therapy, or

ii)	Provocation challenge undertaken to local Trust policy which indicates the presence of positive airway hyperresponsiveness, or

iii)	change in FEV_1_ by ≥ 12% and ≥ 200 mL after acute reversibility testing with 400 μg albuterol or 2.5–5 mg nebulized salbutamol

**Exclusion criteria for people with severe asthma**

People with asthma who met any of the following criteria were excluded from study entry:

- Treatment with intravenous [IV], intramuscular [IM]) or intraarticular corticosteroids within 4 weeks prior to Visit 1 or during the screening period for any reason, including an acute exacerbation event
- A severe asthma exacerbation requiring oral corticosteroids within 4 weeks, defined as an exacerbation of asthma requiring the new administration of oral steroids or an increase of at least 10 mg in their usual oral prednisolone dose within the last 4 weeks – defined from the last day of adjusted prednisolone therapy (such patients could re-screened >4 weeks from the last exacerbation)..
- Infection that meets any of the following criteria:
  - Any infection that resulted in hospital admission for ≥24 hours within 4 weeks prior to Visit 1 or during screening
  - Any infection that required treatment with IV or IM antibiotics within 4 weeks prior to Visit 1 or during screening
  - Any active infection that required treatment with oral antibiotics within 2 weeks prior to Visit 1 or during screening
  - Upper or lower respiratory tract infection within 4 weeks prior to Visit 1 or during screening
  - Antibiotics include any antimicrobial therapy used to treat bacterial, fungal, parasitic, viral, or other infections. Antibiotics prescribed for lung infection prophylaxis would also exclude the patient.
- Active tuberculosis requiring treatment within 12 months prior to Visit 1
  - Patients who have completed treatment for tuberculosis at least 12 months prior to Visit 1 and have no evidence of recurrent disease are permitted.
- Known immunodeficiency, including, but not limited to, HIV infection
- Evidence of acute or chronic hepatitis or known liver cirrhosis
- AST, ALT, or total bilirubin elevation ≥ 2.0 x the upper limit of normal (ULN) during screening
- Clinically significant abnormality on screening electrocardiogram (ECG) or laboratory tests (haematology, serum chemistry, and urinalysis) that, in the opinion of the investigator, may pose an additional risk of bronchoscopy
- History of clinically significant lung disease other than asthma
- Known current malignancy or current evaluation for a potential malignancy
- Unable to safely undergo elective flexible fibreoptic bronchoscopy because of any one of the following:
  - History of allergic reactions to local anesthetics to be used in the procedure
  - History of a clinically significant clotting abnormality, including on Screening Coagulation Panel
  - History of acute myocardial infarction, unstable angina, or other medical conditions that, in the opinion of the investigator, may make the patient unsuitable for elective bronchoscopy
- Other clinically significant medical disease that is uncontrolled despite treatment, that is likely, in the opinion of the investigator, to impact the patient's ability to participate in the study
- Current smoker, former smoker with smoking history of ≥15 pack-years
  - A current smoker was defined as someone who has smoked at least one cigarette per day (or pipe, cigar, or marijuana) for ≥ 30 days within the 12 months prior to Visit 1. A pack-year was defined as the average number of packs of cigarettes per day times the number of years of smoking.

•	Use of a licensed or investigational monoclonal antibody including anti–IL 13, anti-IL-4/IL-13, omalizumab, anti–IL-5, or anti–IL 17, within 6 months or 5 drug half-lives prior to Visit 1 (whichever is longer) or during screening

•	Use of a systemic immunomodulatory or immunosuppressive therapy (other than a monoclonal antibody or corticosteroids [see separate exclusion]) within 3 months or 5 drug half-lives prior to Visit 1 (whichever is longer) or during screening

•	Use of other investigational therapy not described above within 4 weeks or 5 drug half-lives prior to Visit 1 (whichever is longer) or during screening

-	Patients participating in a clinical trial that has not been unblinded should be assumed to have received the active drug

•	Initiation of or change in allergen immunotherapy within 3 months prior to Visit 1 or during screening

•	Receipt of a live attenuated vaccine within 4 weeks prior to Visit 1 or during screening

•	Pregnant or lactating

•	History of bronchial thermoplasty

## Inclusion criteria for healthy volunteers in the Leicester healthy volunteer study (HVS)

Healthy Subjects were required to have no respiratory co-morbidity or clinically important non-respiratory co-morbidity (including allergic rhinitis [perennial or seasonal], atopic dermatitis, food allergy). Participants consented to confirm that they provided accurate information regarding their medical/ surgical history and that a participants General Practitioner could be approached should further confirmation be required.

In addition, the following were required:

1.	Participant was willing and able to give informed consent for participation in the study.

2.	Male or Female, aged 18-65 years at Screening Visit 1

3.	An FEV_1_ of >80% predicted

4.	An FEV_1_/FVC ratio ≥70%

5.	Non-smoker for at least 1 year with <10 pack year smoking history

6.	Participant has clinically acceptable laboratory and ECG at screening (visit 1)

7.	A chest x-ray (arranged at screening (visit 1)) that confirms the absence of clinically significant lung disease unless a chest X-ray or CT scan had been obtained within 12 months before screening (Visit 1) and is available for review by the investigator.

8.	A negative result to skin prick testing with common aeroallergens (house dust mite, grass, tree-mix, cat, dog), or, if a skin prick test to a common aeroallergen is positive, a negative response to challenge with methacholine (defined as PC20 methacholine >16 mg/ml).

9.	Able (in the Investigators opinion) and willing to comply with all study requirements.

10.	Willing to allow his or her General Practitioner and consultant to be notified of participation in the study.

**Exclusion criteria** for the Leicester Healthy volunteer study were similar to those for the RASP study (see above).

**Standard operating procedures**

All centres were experienced at performing research bronchoscopies in asthma. RASP, and Leicester HVS used the same standard operating procedures for tissue collection and processing. These are summarised briefly as follows:

**Bronchoscopy**

Subjects underwent bronchoscopy conducted according to British Thoracic Society (BTS) guidelines (8). Biopsies and brushings were collected from 2^nd^-5^th^ generation bronchi under direct vision as per study procedure manual.

## RNA sequencing

Bronchial biopsies and brushings were collected in RNAlater as per study procedure manual. Total RNA and DNA were isolated from bronchial biopsies and brushings using QIAgen AllPrep DNA/RNA/miRNA Universal Mini Kit per manufacturer’s instructions. Ribosomal RNA was removed using the RiboZero Magnetic Gold kit. RNA sequencing libraries were prepared using the Illumina TruSeq Stranded Total RNA method. Libraries were single-end sequenced. The RNA Sequencing data have been deposited in the Gene Expression Omnibus (GEO) under accession numbers GSE242048 and GSE301357.

For analysis of RNA-seq data, sequences in fastq files (in single and paired ends) were aligned using STAR aligner (version 2.7.1a) to the human reference genome GRCh38; R package (9)*Rsubread* was employed for quantification of reads assigned to genes. RASP and some HVS samples were sequenced using a single-end strategy, which is cost-effective, faster, and sufficient for most gene expression studies but can introduce alignment ambiguity, particularly in regions with overlapping genes on the same strand, as is observed in 10x Genomics data. To mitigate this, we used optimised reference annotations provided by the Pool lab (<https://www.thepoollab.org/resources>), specifically designed to reduce such ambiguities and improve gene quantification accuracy.

## Gene expression analysis

The RNA Sequencing data have been deposited in the Gene Expression Omnibus (GEO) under accession numbers GSE242048 and GSE301357. Raw count data were pre-processed and filtered using the *edgeR* package. For normalisation, Variance Stabilizing Normalization method via *vsn* packages was applied. Differential expression analysis was performed using the *DESeq2* package (10), which models read counts using a negative binomial distribution. Genes with an adjusted p-value (Benjamini-Hochberg false discovery rate, FDR) < 0.05 were considered significantly differentially expressed. Variance stabilising transformation (VST) was applied to normalise count data for downstream analyses. To identify gene signatures of inflammatory biomarkers, we performed differential gene expression analyses using the limma-voom pipeline, adjusting for potential covariates such as age and sex. Each gene's association with biomarkers was modelled as a continuous linear predictor of biomarker levels.

Data integration exploring the ICS effect on asthma gene transcription asthma was performed initially using ComBat from the *sva* package to remove batch effects (11). To confirm robustness against batch effects and variability, differential expression was also assessed using the non-parametric, rank-based method implemented in the *RankProd* package (12). Rank product analysis (RPA) multiplies the ranks of features across replicates, and calculates a significance score robust against noise. It is generally more sensitive with higher statistical power for detecting DEGs in these types of datasets and robust to batch effects.

Gene Ontology (GO) enrichment analysis was performed using the *clusterProfiler* package (13), with gene sets obtained from the Gene Ontology Consortium. Gene set enrichment analysis (GSEA) (14) was conducted to investigate pathway-level changes across T2-phenotypes, using curated immune-related pathways sourced from the ImmPort database (15). Gene Set Variation Analysis (GSVA)(16) was used to evaluate the enrichment of gene signatures including T2 cytokine- and IL-17-dependent gene signatures. Weighted Gene Co-expression Network Analysis (WGCNA) (17) was employed to identify modules of co-expressed genes associated with the T2-phenotype. Module-trait relationships were assessed by correlating module eigengenes with clinical metadata. For unsupervised clustering of the gene expression matrix, K-means clustering was applied using the *ClusterGVis* package (18), with the optimal number of clusters determined by the silhouette method. In the transcription factor enrichment analysis, transcription factor (TF) activity inference was performed using DoRothEA (19), which integrates curated regulons and gene expression data to estimate TF activity scores.

To estimate the relative proportions of cell subsets in each sample, we performed cell deconvolution analysis on bronchial biopsy transcriptomic data, using single-cell signatures from the Travaglini lung cell atlas (20). This molecular cell atlas provides a powerful bioinformatic resource as it includes individually annotated samples taken from human proximal airways, and from distal airways, as well as lung parenchyma and alveoli, and included cells from an individual with mild adult-onset asthma.

## Statistical analysis

Categorical variables are presented as frequencies and percentages, and continuous variables were reported as means with mean (standard error mean [SEM]) or median (interquartile range [IQR]), depending on distribution. Comparisons of multiple groups were conducted using Chi-square tests for categorical variables, and ANOVA or Kruskal-Wallis tests for continuous variables, as appropriate. Correlation between clinical characteristics were assessed using Spearman’s rank correlation coefficient. For assessment of differential gene expression, a log2 fold change ≥0.5 was considered biologically relevant. All statistical analyses were performed using R software (version 4.4.0), with significance set at p < 0.05.

For transcriptomic data, all differential expression models were adjusted for age, sex, and previous smoking status, and included consistently across all DESeq2 analyses. There were no missing data in the clinical covariates. For clinical data, missing data was minimal and handled using complete-case analysis.

## Validation using the U-BIOPRED bronchoscopy cohort

The Unbiased Biomarkers for the Prediction of Respiratory Disease Outcomes (U-BIOPRED) study was a multi-centre, pan-European asthma study conducted at 16 clinical centres across 11 European countries (21). The U-BIOPRED study was approved by the ethics committees in each participating country and was registered on ClinicalTrials.gov (NCT01982162). In this study, only adult non-current smoking participants who have undergone bronchoscopy were included. Patients with severe asthma were classified according to the RASP biomarker cut-offs, with the exception that the T2-intermediate group were those not meeting the biomarker definitions for T2-high or T2-low.

Demographic, clinical, lung function (spirometry and body plethysmography), physiological data, medications, comorbidities, and severe events were collected as previously described (21, 22). Cell counts from blood, induced sputum, and biopsies were obtained, along with a range of biomarkers, including serum IgE, serum periostin, fractional exhaled nitric oxide (FeNO) and C-reactive protein (CRP) as previously described (23).

For analysis, we extracted data from the tranSMART knowledge platform (<https://ubiopred.dsi.ic.ac.uk/transmart/>) using the previously generated count matrix. Transcriptomic analyses were as described for RASP above.

# SUPPLEMENTARY RESULTS

**Clinical characteristics**

We assessed correlations between clinical parameters and as expected from other studies observed strong positive correlations between FEV_1_% predicted and forced expiratory ratio (FER). There were moderate positive correlations between BMI and ACQ-5 symptom score (**Figure S2A**), and between FeNO and blood eosinophils (r = 0.55, p 2.2x10^-5^, **Figure S2B**). Negative correlations were observed between age and IgE, and age and FER (**Figure S2A**).

**Cell deconvolution analysis**

In cell deconvolution analysis on bronchial biopsy transcriptomic data we compared with single-cell signatures from a lung cell atlas by Travaglini *et al* (20). This molecular cell atlas provides a powerful bioinformatic resource as it includes individually annotated samples taken from human proximal airways, and from distal airways, as well as lung parenchyma and alveoli, and includes cells from an individual with mild adult-onset asthma.

We found that asthma was associated with enrichment of activated NK cells, but reduced total NK cells, plasmacytoid and myeloid DCs, increased goblet cells, and reduced lung B cells, naïve and effector memory CD4+ and naïve CD8+ T cells, classical monocytes, adventitial fibroblasts, and natural killer T cells (**Table S4, Figure S3A**).

## T2-high versus T2-low asthma

Confirmation of the findings from DESeq2 was demonstrated using a non-parametric rank product analysis (RPA) approach and shown in **Table S7**. RPA multiplies the ranks of features across replicates, and calculates a significance score robust against noise, and is generally more sensitive with higher statistical power for detecting DEGs in these types of datasets. T2-high asthma was associated with prominent upregulation of: inducible nitric oxide synthase (NOS2); more epithelial barrier genes (PRB1, PRB2, PRB4, PRB3, PRB11, PRB31,PRB41); more keratin genes (KRT6A, KRT6A1, KRT4, KRT24, KRT13, KRT131, KRT14, KRT141); the pro-inflammatory activator protein-1 transcription factors FOS and FOSB; the immunoregulatory matricellular protein CCN1 which promotes neutrophil efferocytosis and IL-8 secretion(24); the early growth response transcription factors EGR1, EGR2 and EGR3 which are implicated in induction of allergic responses; transcobalamin-1 (TCN1), a gene we previously found highly-upregulated in severe asthma(25) and which is highly expressed in airway Th2 cells(26). Upregulated biological process GO terms in T2-high asthma were related to cell proliferation and histone modifications (**Table S9**). There was relative downregulation of biological processes related to cilial function, with the terms ‘cilium organisation’, ‘cilium movement’ and ‘axoneme assembly’ each amongst the 5 least expressed processes (**Table S9**). Deconvolution using the single-cell atlas in T2-high asthma predicted an increase in NK cells, lung basal cells, differentiating basal cells, bronchial vessel cells, naïve CD4+ T cells and goblet cells (**Table S10**).

## T2-low asthma

T2-low asthma was associated with ASM including the ryanodine receptor (RYR3), alpha smooth muscle actin (ACT2A) and the voltage gated K^+^ channel KCNA1 (Kv1.1)(**Table S7**). There was also marked upregulation of several surfactant proteins, including serum amyloid A1 and A2 (SAA1, SAA2), BPIFA1 (PLUNC) and surfactant protein A2 (SFTPA2), which have anti-inflammatory and innate immunomodulatory activity, and of the Th1 polarising lymphocyte chemokine CXCL9 (MIG). Rank product analyses showed T2-low asthma was associated with upregulation specifically of signatures for Treg and adenosine-activated mast cells (**Table S8**). There was relative upregulation of biological processes related to cilial function, with the terms ‘cilium organisation’, ‘cilium movement’ and ‘axoneme assembly’ each amongst the 5 most enriched processes (**Table S9**). Cell deconvolution showed an increase in cells annotated as mast cells or basophils in T2-low asthma (**Table S10**), although this did not reflect the expression of the mast cell-specific genes such as tryptases, MS4A2 and CPA3 which were increased in the epithelium of T2-high patients.

## T2-intermediate asthma

T2-intermediate asthma was associated with upregulation specifically of signatures for leukocyte responses to viruses including virally activated neutrophils (**Table S12**). There was relative upregulation of biological processes related to activated leukocytes, lymphocytes and adaptive T cell responses (**Table S11**). Cell deconvolution showed greatest increases in cells annotated as monocytes, naïve lung CD8 T cells, basophil/mast cells, macrophages and NK cells (**Table S13**).

**Weighted Gene Network Correlation Analysis**

To explore how specific modules of genes related to individual clinical traits we used weighted gene co-expression network analysis (WGCNA) (**Figure S6**). The most significant module-trait relationships were between the following modules: T2-intermediate asthma was associated positively with the ‘*Immune response module*’ whose GO terms included ‘immune response-regulating signaling pathway’, ‘leukocyte mediated immunity’, ‘regulation of effector process’, ‘leukocyte proliferation’ and ‘lymphocyte proliferation’ (**Figure S6E**); T2-intermediate asthma was also associated positively with a ‘*Pathogen defence module*’ whose GO terms included responses to viruses, symbionts and type II interferon. This module was associated negatively with FeNO levels, consistent with a mutually exclusive association we recently described between FeNO and *Haemophilus influenzae*(27). A ‘*Development and metabolism*’ module was associated positively with male sex and negatively with symptoms (ACQ-5); an ‘*Organic metabolism*’ module, driven by the GO term ‘xenobiotic metabolic process’ was highly associated with female sex.

**Validation in the U-BIOPRED cohort**

Despite U-BIOPRED having a different patient recruitment strategy, and using microarrays rather than RNAseq for gene expression analysis, there were excellent correlations when comparing the log2 fold change for asthma versus healthy (no ICS) from RASP and U-BIOPRED, in both biopsies and brushes for asthma overall (**Figure S7**), and for the T2-high versus T2-low and T-2 intermediate analyses (**Figures S8 and S9**). There were relatively few discordant genes. U-BIOPRED demographic data are presented in **Table S14**.

# SUPPLEMENTARY DISCUSSION

A combination of T1- and T2-dependent inflammation is described by T1- and T2-dependent transcriptional signatures in 8.5% of patients with severe asthma(28) or by cellular sputum cytokine content(29). We found that IFNγ-dependent genes and IFNγ signalling pathways were enriched in those with T2-low disease. This may in part be due to the compartment sampled (airway epithelium rather than sputum or BAL), but also the clear a priori criteria for separating T2-high and T2-low disease.

We observed distinct gene modules associated to different extents with distinct type 2 biomarkers: fractional exhaled nitric oxide and blood eosinophil counts (**Figure S8**). Whilst these two clinically-accessible parameters are generally considered together as type 2 biomarkers, the strength of their correlation in this dataset is only moderate (Spearman’s R=0.55, **Figure S1**, so we consider they represent two different aspects of type 2 biology. Elsewhere we have shown FeNO and blood eosinophil counts provide independent prognostic and predictive information regarding asthma exacerbations(30, 31). Based on cross-sectional analysis of inflammatory proteins in sputum and serum from patients with severe asthma we argue that serum IL-5 alone correlates with the magnitude of the systemic pool of peripheral blood eosinophils which are the main immune effector cell during exacerbations (conceptualised as ‘bombs’), whilst FeNO correlates with almost all other components of the chemotactic pull to their airways (conceptualised as the strength of the mucosal ‘magnet’)(32).

The gene module traits we observe by WGCNA of bronchial biopsies are consistent with this paradigm: notably FeNO is most strongly inversely related to a ‘Pathogen defence module’, reflective of the well-known inverse, counter-regulatory relationship between type 1 and type 2 inflammation within mucosal surfaces. By contrast this association was not observed with blood eosinophils, which were rather inversely associated with an organic metabolism module. These differences can be further dissected at the level of individual genes (**Figure 3A**) where it is apparent that some genes such as POSTN (periostin) and TAC1 (encoding the neurotransmitter substance P) were significantly associated with FeNO only, whilst others like EDN2 (endothelin 2) and FETUB were associated only with blood eosinophils. FETUB encodes fetuin B, an inhibitor of neprilysin, and therefore its expression enhances airway signalling via neuropeptides such as CGRP(33). These different associations support our proposed two-compartment model of airway inflammation. Moreover, the heterogeneity may provide clues as to why some patients respond more to biologics targeting the IL-5-IL5Ra axis, whilst others are more responsive to those targeting epithelial alarmins and the IL4Ra receptor, and also underscore the currently under-researched field of neuropeptides in asthma biology and their potential as future therapeutic targets.

Bronchial brushes are essentially a subset of the biopsies, so while we would expect an overall positive correlation, there will inevitably some discrepancies, as evident in table S1. There was nevertheless a strong positive correlation.

Mast cell-specific genes and gene signatures for resting and FceRI-activated mast cells were downregulated in asthma bronchial biopsies in asthma *versus* health, in keeping with the reduced mast cell numbers in the lamina propria identified previously in this and other cohorts (e.g. U-BIOPRED) of people with severe asthma(1, 34). In contrast in epithelial brushes, mast cell-specific genes and the same mast cell signatures were increased in T2-high asthma and some patients with T2-intermediate asthma, but not T2-low asthma. This is surprising considering that mast cells were rarely seen in the airway epithelium in this and other cohorts using immunohistochemistry, but in keeping with elevated concentrations of PGD2 in the sputum of these patients(1), and elevated mast cell specific proteases in sputum(35), elevated mast cell gene expression signatures(36) and the very high levels of total and activated tryptase levels in bronchosorption samples(37) in previous studies. The discrepancy between the immunohistochemistry data (reduced cells), and the elevated sputum mediators and gene expression in severe asthma may be due to extensive mast cell degranulation, rendering mast cell identification by immunohistochemistry sub-optimal. While T2-low asthma did not express these classic mast cell signatures, there was evidence of an adenosine–activated mast cell signature in T2 low asthma identified by GSEA, and elevated PGD2 in the sputum of these patients(1). Overall, these and previous data suggest that mast cell activation is present across T2-high and T2-low asthma.

Several genes implicated in the maintenance and repair of epithelial barrier function were upregulated in the biopsies of T2-high compared to T2-low asthma. Several claudins involved directly in tight junction formation (CLDN3/8/16; SCGB1A1) were downregulated in T2-high asthma. PRB1 and 2 are members of the BstNI subfamily of salivary proline-rich proteins, and were amongst the most significantly upregulated genes (tables S6 and S7). Both PRB1 and PRB2 are precursors that are cleaved into multiple basic proline-rich proteins. Their activity remains very poorly characterised, but it has been proposed that PRB1 contributes to barrier function in the cornea(38) . There is a report of elevated PRB1 protein in the sputum of T2 high asthmatics, which supports our findings here(39). ROR1 was elevated in the airway epithelial brushes in T2-high asthma compared to T2-low asthma (table S7), and is implicated in the process of epithelial-mesenchymal transition(40), a recognised feature of asthma(41). Both PRD proteins and ROR1, if involved in barrier function in the airway, are likely to affect barrier function indirectly.

The asthma Origins and Remission Study (ARMS) recently examined bronchial brush gene expression by RNAseq in 26 people with asthma who were able to cease ICS/LABA use for 6 weeks before undergoing a bronchial brush and paired nasal brush(42). In the bronchial brushes there were 51 DEGS in asthma compared to healthy controls, 40 upregulated, 11 downregulated. As most of the patients had mild/moderate asthma in ARMS and most had been treated with ICS previously but were then able to withhold ICS/LABA for 6 weeks, it is difficult to draw major conclusions from a comparison ARMS with RASP. The molecular effects of ICS in ARMS would have been expected to have disappeared but some residual ICS effect may have persisted. Nevertheless, it is noteworthy that 27/40 genes upregulated in ARMS brushes were upregulated in RASP brushes, and 4/11 genes downregulated in ARMS brushes were downregulated in RASP brushes. The overall correlation for the significantly co-regulated genes was r=0.88, p=5.04E-11.

Many genes with altered expression in the airways of people with asthma compared to healthy controls, were concordantly dysregulated by ICS in healthy people. Identifying this ICS treatment effect is clearly important as molecular expression might otherwise be mistakenly attributed to asthma pathophysiology, and might be inappropriately targeted for drug development. Examples of genes upregulated both in asthma and by ICS include ALOX-15B, EREG and NNMT. The products of these 3 genes have both beneficial and detrimental effects for health depending on the context of their expression. ALOX-15B encodes 15-lipoxygenase-2 and generates both pro-inflammatory and pro-resolving mediators from polyunsaturated fatty acids (reviewed in (43)). It is implicated in the development of macrophage-dependent inflammation in atherosclerosis, but is also a tumour suppressor, and potentially anti-inflammatory. EREG encodes epiregulin, a ligand for the epidermal growth factor receptor implicated in both physiological tissue repair and pathological fibrosis(44). NNMT encodes nicotinamide N-methyltransferase, an enzyme involved in DNA methylation, and is linked to tissue fibrosis(45). The upregulation of ALOX-15B, EREG, NNMT and other genes by ICS suggests that their upregulation in asthma may primarily be a beneficial corticosteroid effect, but it is plausible that not all the effects of corticosteroids in the airways are beneficial, raising the possibility that in some individuals, upregulation of genes like ALOX-15B, EREG and NNMT might be harmful as well as protective, depending on the individual expression level and activity.

Our data confirm that those people with the highest T2 biomarkers have the highest T2 molecular activity, and supports the clinical observation that the higher the T2 biomarkers, the greater the efficacy of biologics targeting the T2 molecular pathway. Tezepelumab shows greater clinical efficacy in those with lower T2 biomarker levels compared to biologics targeting IL-5/IL-5Rα or IL-4Rα, but nevertheless is most effective in those with high T2 biomarkers. The mechanisms through which tezepelumab exerts benefit in T2-low disease remain unclear, but inhibition of the effects of TSLP on ASM , mast cells and broader upstream suppression of epithelial alarmin signaling may be contributing factors(46, 47).

# SUPPLEMENTARY REFERENCES

1.	Khalfaoui L, Symon FA, Couillard S, Hargadon B, Chaudhuri R, Bicknell S, et al. Airway remodelling rather than cellular infiltration characterizes both type2 cytokine biomarker-high and -low severe asthma. Allergy. 2022;77(10):2974-86.

2.	Marchi E, Hinks TSC, Richardson M, Khalfaoui L, Symon FA, Rajasekar P, et al. The effects of inhaled corticosteroids on healthy airways. Allergy. 2024;79(7):1831-43.

3.	Heaney LG, Busby J, Bradding P, Chaudhuri R, Mansur AH, Niven R, et al. Remotely Monitored Therapy and Nitric Oxide Suppression Identifies Nonadherence in Severe Asthma. Am J Respir Crit Care Med. 2019;199(4):454-64.

4.	Boddy CE, Naveed S, Craner M, Murphy AC, Siddiqui S, Bradding P. Clinical Outcomes in People with Difficult-to-Control Asthma Using Electronic Monitoring to Support Medication Adherence. J Allergy Clin Immunol Pract. 2020.

5.	McNicholl DM, Stevenson M, McGarvey LP, Heaney LG. The utility of fractional exhaled nitric oxide suppression in the identification of nonadherence in difficult asthma. Am J Respir Crit Care Med. 2012;186(11):1102-8.

6.	Heaney LG, Busby J, Hanratty CE, Djukanovic R, Woodcock A, Walker SM, et al. Composite type-2 biomarker strategy versus a symptom-risk-based algorithm to adjust corticosteroid dose in patients with severe asthma: a multicentre, single-blind, parallel group, randomised controlled trial. Lancet Respir Med. 2020.

7.	Choy DF, Hart KM, Borthwick LA, Shikotra A, Nagarkar DR, Siddiqui S, et al. TH2 and TH17 inflammatory pathways are reciprocally regulated in asthma. Sci Transl Med. 2015;7(301):301ra129.

8.	Du Rand IA, Blaikley J, Booton R, Chaudhuri N, Gupta V, Khalid S, et al. British Thoracic Society guideline for diagnostic flexible bronchoscopy in adults: accredited by NICE. Thorax. 2013;68 Suppl 1:i1-i44.

9.	Foundation TR. The R Project for Statistical Computing [Available from: <http://www.r-project.org/index.html>.

10.	Love MI, Huber W, Anders S. Moderated estimation of fold change and dispersion for RNA-seq data with DESeq2. Genome Biology. 2014;15(12):550.

11.	Johnson WE, Li C, Rabinovic A. Adjusting batch effects in microarray expression data using empirical Bayes methods. Biostatistics. 2007;8(1):118-27.

12.	Hong F, Breitling R, McEntee CW, Wittner BS, Nemhauser JL, Chory J. RankProd: a bioconductor package for detecting differentially expressed genes in meta-analysis. Bioinformatics. 2006;22(22):2825-7.

13.	Yu G, Wang L-G, Han Y, He Q-Y. clusterProfiler: an R package for comparing biological themes among gene clusters. Omics: a journal of integrative biology. 2012;16(5):284-7.

14.	Subramanian A, Tamayo P, Mootha VK, Mukherjee S, Ebert BL, Gillette MA, et al. Gene set enrichment analysis: a knowledge-based approach for interpreting genome-wide expression profiles. Proceedings of the National Academy of Sciences. 2005;102(43):15545-50.

15.	Bhattacharya S, Andorf S, Gomes L, Dunn P, Schaefer H, Pontius J, et al. ImmPort: disseminating data to the public for the future of immunology. Immunologic research. 2014;58:234-9.

16.	Hänzelmann S, Castelo R, Guinney J. GSVA: gene set variation analysis for microarray and RNA-seq data. BMC Bioinformatics. 2013;14:7.

17.	Langfelder P, Horvath S. WGCNA: an R package for weighted correlation network analysis. BMC bioinformatics. 2008;9:1-13.

18.	Zhang J. ClusterGVis: One-step to Cluster and Visualize Gene Expression Matrix. <https://github.com/junjunlab/ClusterGVis> 2022 [

19.	Garcia-Alonso L, Holland CH, Ibrahim MM, Turei D, Saez-Rodriguez J. Benchmark and integration of resources for the estimation of human transcription factor activities. Genome Res. 2019;29(8):1363-75.

20.	Travaglini KJ, Nabhan AN, Penland L, Sinha R, Gillich A, Sit RV, et al. A molecular cell atlas of the human lung from single-cell RNA sequencing. Nature. 2020;587(7835):619-25.

21.	Shaw DE, Sousa AR, Fowler SJ, Fleming LJ, Roberts G, Corfield J, et al. Clinical and inflammatory characteristics of the European U-BIOPRED adult severe asthma cohort. Eur Respir J. 2015;46(5):1308-21.

22.	Wilson SJ, Ward JA, Pickett HM, Baldi S, Sousa AR, Sterk PJ, et al. Airway Elastin is increased in severe asthma and relates to proximal wall area: histological and computed tomography findings from the U‐BIOPRED severe asthma study. Clinical & Experimental Allergy. 2021;51(2):296-304.

23.	Kuo CS, Pavlidis S, Loza M, Baribaud F, Rowe A, Pandis I, et al. T-helper cell type 2 (Th2) and non-Th2 molecular phenotypes of asthma using sputum transcriptomics in U-BIOPRED. Eur Respir J. 2017;49(2).

24.	Jun JI, Kim KH, Lau LF. The matricellular protein CCN1 mediates neutrophil efferocytosis in cutaneous wound healing. Nature communications. 2015;6:7386.

25.	Singhania A, Wallington JC, Smith CG, Horowitz D, Staples KJ, Howarth PH, et al. Multitissue Transcriptomics Delineates the Diversity of Airway T Cell Functions in Asthma. Am J Respir Cell Mol Biol. 2018;58(2):261-70.

26.	Ma J, Tibbitt CA, Georen SK, Christian M, Murrell B, Cardell LO, et al. Single-cell analysis pinpoints distinct populations of cytotoxic CD4(+) T cells and an IL-10(+)CD109(+) T(H)2 cell population in nasal polyps. Sci Immunol. 2021;6(62).

27.	Jabeen MF, Sanderson ND, Tine M, Donachie G, Barber C, Azim A, et al. Species-level, metagenomic and proteomic analysis of microbe-immune interactions in severe asthma. Allergy. 2024.

28.	Fahy JV, Jackson ND, Sajuthi SP, Pruesse E, Moore CM, Everman JL, et al. Type 1 Immune Responses Related to Viral Infection Influence Corticosteroid Response in Asthma. Am J Respir Crit Care Med. 2025;211(2):194-204.

29.	Marks M, Steele C, Moore WC, Meyers DA, Rector B, Ampleford E, et al. Tumour necrosis factor-related apoptosis-inducing ligand (TRAIL) and receptors in type 1, type 2 and type 17 inflammation in cross-sectional asthma study. Thorax. 2020;75(9):808-11.

30.	Couillard S, Laugerud A, Jabeen M, Ramakrishnan S, Melhorn J, Hinks T, Pavord I. Derivation of a prototype asthma attack risk scale centred on blood eosinophils and exhaled nitric oxide. Thorax. 2022;77(2):199-202.

31.	Meulmeester FL, Mailhot-Larouche S, Celis-Preciado C, Lemaire-Paquette S, Ramakrishnan S, Wechsler ME, et al. Inflammatory and clinical risk factors for asthma attacks (ORACLE2): a patient-level meta-analysis of control groups of 22 randomised trials. Lancet Respir Med. 2025;13(6):505-16.

32.	Couillard S, Pavord ID, Heaney LG, Petousi N, Hinks TSC. Sub-stratification of type-2 high airway disease for therapeutic decision-making: A 'bomb' (blood eosinophils) meets 'magnet' (FeNO) framework. Respirology. 2022;27(8):573-7.

33.	Sun P, Hua Q, Fu H, Yao L, Yuan X, Li Q, et al. Epithelial FETUB-mediated the inhibition of NEP activity aggravates asthma. Int Immunopharmacol. 2025;152:114397.

34.	Wilson SJ, Ward JA, Sousa AR, Corfield J, Bansal AT, De Meulder B, et al. Severe asthma exists despite suppressed tissue inflammation: findings of the U-BIOPRED study. Eur Respir J. 2016;48(5):1307-19.

35.	Hinks TS, Zhou X, Staples KJ, Dimitrov BD, Manta A, Petrossian T, et al. Innate and adaptive T cells in asthmatic patients: Relationship to severity and disease mechanisms. J Allergy Clin Immunol. 2015;136(2):323-33.

36.	Tiotiu A, Badi Y, Kermani NZ, Sanak M, Kolmert J, Wheelock CE, et al. Association of Differential Mast Cell Activation to Granulocytic Inflammation in Severe Asthma. Am J Respir Crit Care Med. 2021.

37.	Rhee H, Henderson LM, Bauer RN, Wong K, Staton TL, Choy DF, et al. Airway tryptase levels inform the lack of clinical efficacy of the tryptase inhibitor MTPS9579A in asthma. Allergy. 2024;79(11):2993-3004.

38.	Qi X, Ren SW, Zhang F, Wang YQ. The involvement of proline-rich protein Mus musculus predicted gene 4736 in ocular surface functions. Int J Ophthalmol. 2016;9(8):1121-6.

39.	Chen F, Liang Y, Zeng Z, Du L, Xu C, Guo Y, Xie C. Association of increased basic salivary proline-rich protein 1 levels in induced sputum with type 2-high asthma. Immun Inflamm Dis. 2022;10(4):e602.

40.	Jung MS, Choi WY, Zhang W, Barrera FN, Perkins RS. The Role of Receptor Tyrosine Kinase-like Orphan Receptor 1 (ROR1) in Cancer Stem Cell Signaling. Int J Mol Sci. 2025;26(16).

41.	Pain M, Bermudez O, Lacoste P, Royer PJ, Botturi K, Tissot A, et al. Tissue remodelling in chronic bronchial diseases: from the epithelial to mesenchymal phenotype. Eur Respir Rev. 2014;23(131):118-30.

42.	Wen H, Kole T, Carpaij OA, Karp T, Guryev V, Faiz A, et al. Nasal Airway Transcriptome Reflects Selected Asthma-Associated Gene Signatures in the Lower Airways. Allergy. 2026.

43.	Benatzy Y, Palmer MA, Brune B. Arachidonate 15-lipoxygenase type B: Regulation, function, and its role in pathophysiology. Front Pharmacol. 2022;13:1042420.

44.	Ezaddoustdar A, Kalina D, Bielohuby M, Boehm M, Wygrecka M. dEREGulated pathways: unraveling the role of epiregulin in skin, kidney, and lung fibrosis. Am J Physiol Cell Physiol. 2025;328(2):C617-C26.

45.	Lai SY, Ni XL, Bi SZ, Sun WD, Liu A, Zhang CY, Li JH. NNMT as a therapeutic target in fibrosis: Insights from the heart, liver, kidneys, and lungs. Differentiation. 2026;149:100959.

46.	Smelter DF, Sathish V, Thompson MA, Pabelick CM, Vassallo R, Prakash YS. Thymic stromal lymphopoietin in cigarette smoke-exposed human airway smooth muscle. J Immunol. 2010;185(5):3035-40.

47.	Gauvreau GM, O'Byrne PM, Boulet LP, Wang Y, Cockcroft D, Bigler J, et al. Effects of an anti-TSLP antibody on allergen-induced asthmatic responses. N Engl J Med. 2014;370(22):2102-10.

# SUPPLEMENTARY TABLES

## Supplementary Table S1. All differentially expressed genes between asthma versus health in bronchial biopsies and brushings.

All differentially expressed genes between asthma *versus* healthy controls receiving ICS; asthma *versus* healthy controls not receiving ICS.

Please see accompanying Excel file - Table S1

## Supplementary Table S2. Gene Ontology Biological Process pathways enriched in asthma *versus* healthy controls receiving ICS in bronchial biopsies.

Gene Ontology (GO) Biological Process pathways significantly enriched between asthma patients and healthy controls receiving inhaled corticosteroids were identified using rank product analysis. Pathways shown are ordered by P value. Under figure *x* axis represents approximate gene ranks. NES, normalised enrichment score.

## Supplementary Table S3. Immune response pathways enriched in asthma *versus* healthy controls receiving ICS in bronchial biopsies.

Immune response pathways significantly enriched between asthma patients and healthy controls receiving inhaled corticosteroids were identified using rank product analysis. Pathways shown are ordered by P value. Under figure *x* axis represents approximate gene ranks. NES, normalised enrichment score.

## Supplementary Table S4. Lung cell type pathways enriched in asthma *versus* healthy controls receiving ICS in bronchial biopsies.

Lung cell type pathways significantly enriched between asthma patients and healthy controls receiving inhaled corticosteroids were identified using rank product analysis. Pathways shown are ordered by P value. Under figure x axis represents approximate gene ranks. NES, normalised enrichment score.

## Supplementary Table S5. The log2 fold change for asthma versus health, and healthy versus healthy + ICS, for all detected genes in asthma. This was used to generate figure 2

Please see accompanying Excel file -Table S5

## Supplementary Table S6. All differentially expressed genes between T2-high asthma versus other phenotypes in bronchial biopsies and brushings.

All differentially expressed genes between T2-high asthma *versus* T2-low asthma; between T2-high asthma *versus* T2-intermediate asthma; in biopsies and in brushings. Differential expression analysis was performed using the *DESeq2* package.

Please see accompanying Excel file -Table S6

## Supplementary Table S7. Rank product analysis of differentially expressed genes between T2-high asthma *versus* other phenotypes in bronchial biopsies and brushings.

All differentially expressed genes between T2-high asthma *versus* T2-low asthma; between T2-high asthma *versus* T2-intermediate asthma; in biopsies and in brushings. Differential expression was assessed using the non-parametric, rank-based method implemented in the *RankProd* package

Please see accompanying Excel file -Table S7

Supplementary Table S8. Immune response pathways enriched between T2-high and T2-low asthma in bronchial biopsies. Immune response pathways significantly enriched between T2-high and T2-low asthma were identified using rank product analysis. Pathways shown are ordered by P value. Under figure x axis represents approximate gene ranks. NES, normalised enrichment score.

## Supplementary Table S9. Gene Ontology Biological Process pathways enriched between T2-high and T2-low asthma in bronchial biopsies.

Gene Ontology Biological Process pathways significantly enriched between T2-high and T2-low asthma were identified using rank product analysis. Pathways shown are ordered by P value. Under figure *x* axis represents approximate gene ranks. NES, normalised enrichment score.

## **Supplementary Table S10.** Lung cell type pathways enriched between T2-high and T2-low asthma in bronchial biopsies.

Lung cell type pathways significantly enriched between T2-high and T2-low asthma were identified using rank product analysis. Pathways shown are ordered by P value. Under figure x axis represents approximate gene ranks. NES, normalised enrichment score.

## Supplementary Table S11. Gene Ontology Biological Process pathways between T2-high and T2-intermediate asthma in bronchial biopsies.

Gene Ontology Biological Process pathways significantly enriched between T2-high and T2-intermediate asthma were identified using rank product analysis. Pathways shown are ordered by P value. Under figure *x* axis represents approximate gene ranks. NES, normalised enrichment score.

## Supplementary Table S12. Immune response pathways enriched between T2-high and T2-intermediate asthma in bronchial biopsies.

Immune response pathways significantly enriched between T2-high and T2-low asthma were identified using rank product analysis. Pathways shown are ordered by P value. Under figure *x* axis represents approximate gene ranks. NES, normalised enrichment score.

## **Supplementary Table S13**. Lung cell type pathways enriched between T2-high and T2-intermediate asthma in bronchial biopsies.

Lung cell type pathways significantly enriched between T2-high and T2-intermediate asthma were identified using rank product analysis. Pathways shown are ordered by P value. Under figure x axis represents approximate gene ranks. NES, normalised enrichment score.

## Supplementary table S14. Clinical characteristics of participants in the U-BIOPRED cohort.

| **Characteristics** | **T2-high (n=8)** | **T2-intermediate (n=23)** | **T2-low (n=15)** | **Healthy controls (n=26)** | **p-value** |
| --- | --- | --- | --- | --- | --- |
| Demographics |  |  |  |  |  |
| Age (years) | 61 [49, 65]^*^ | 52 [46, 60]^*^ | 45 [29, 56] | 37 [25, 53] | 0.02 |
| Age at diagnosis (years) | 40 [28, 44]^§^ | 14 [5, 38] | 3.00 [2, 11] | NA | 0.07 |
| Race, Caucasian | 7 (88%) | 22 (96%) | 15 (100%) | 20 (77%) | 0.1 |
| BMI (kg·m^-2^) | 26.6 ± 3.2^#^ | 31.2 ± 5.2^***^ | 31.7 ± 7.1^***^ | 25.0 ± 3.5 | <0.001 |
| Ex-smokers | 2 (25%) | 5 (22%) | 5 (33%) | 4 (15%) | 0.6 |
| Smoking history (pack-years) | 3.5 [2.8, 4.3] | 22.5 [1.5, 33.0] | 5.5 [3.0, 20.0] | 0.5 [0.2, 1.3] | 0.2 |
| Lung function |  |  |  |  |  |
| FEV_1_ pre-bronchodilator (% pred) | 58.4 [49.0, 67.8]^***/§§^ | 62.3 [51.7, 75.5]^***/§§§^ | 88.9 [76.9, 98.6] | 99.0 [90.7, 110.1] | <0.001 |
| FEV_1_/FVC pre-bronchodilator (%) | 68.6 [56.0, 71.4]^***^ | 66.6 [51.4, 70.6]^***/§^ | 76.9 [63.0, 82.0] | 79.6 [75.5, 81.9] | <0.001 |
| Symptoms and exacerbations |  |  |  |  |  |
| ACQ-5 | 1.9 [1.4, 2.6] | 2.2 [1.1, 3.0] | 1.4 [0.9, 1.8] | NA | 0.2 |
| Exacerbations (past year) | 1.5 [0.0, 3.8] | 2.0 [0.5, 3.0] | 2.5 [1.3, 4.0] | NA | 0.4 |
| Severe exacerbations (past year) | 0.5 [0.0, 2.3] | 1.0 [0.0, 2.0] | 2.0 [0.3, 4.0] | NA | 0.4 |
| Biomarkers |  |  |  |  |  |
| IgE (IU/ml) | 107 [43, 295]^*^ | 250 [98, 687]^***/§^ | 69 [18, 243] | 23 [8, 92] | <0.001 |
| FeNO, ppb | 67 [47, 92]^***/§§§^ | 35 [22, 44]^##/§§^ | 21 [15, 23] | 24 [15, 31] | <0.001 |
| Blood and sputum cells |  |  |  |  |  |
| Blood eosinophils, cells/μL | 0.35 [0.30, 0.52]^***/§§§^ | 0.22 [0.15, 0.40]^**/§^ | 0.10 [0.10, 0.20] | 0.10 [0.10, 0.14] | <0.001 |
| Sputum eosinophils, % | 11.91 [7.17, 22.03]^**/§§^ | 3.62 [1.08, 18.37]^**/§^ | 0.38 [0.05, 0.82] | 0.20 [0.00, 0.62] | <0.001 |
| Sputum neutrophils, % | 54.3 [51.9, 63.8]^*^ | 45.7 [35.9, 53.6] | 61.9 [46.5, 75.3]^*^ | 32.7 [14.2, 44.2] | 0.04 |
| Medications |  |  |  |  |  |
| ICS | 8 (100%) | 23 (100%) | 15 (100%) | NA | NA |
| OCS | 5 (63%)^#/§^ | 11 (48%) | 7 (47%) | NA | 0.02 |
| OCS dose, prednisolone (equ.) mg | 8.3 ± 2.4 | 13.8 ± 9.5 | 17.5 ± 9.4 | NA | 0.3 |
| SABA | 8 (100%) | 20 (87%) | 13 (87%) | NA | 0.6 |
| LABA | 8 (100%) | 23 (100%) | 15 (100%) | NA | NA |
| Antibiotic | 1 (13%) | 5 (22%) | 3 (20%) | NA | 0.9 |
| LAMA | 1 (13%) | 7 (30%) | 6 (40%) | NA | 0.4 |
| Omalizumab | 0 (0%) | 2 (9%) | 2 (13%) | NA | 0.6 |
| LTRA | 4 (50%) | 11 (48%) | 12 (80%) | NA | 0.1 |
| Severe events |  |  |  |  |  |
| Endotracheal intubation | 0 (0%) | 3 (13%) | 3 (20%) | NA | 0.4 |
| ICU admission | 1 (13%) | 5 (22%) | 3 (20%) | NA | 0.9 |
|  |  |  |  |  |  |

For continuous variables, the ANOVA or the Kruskal–Wallis test was used across applicable groups. For categorical variables, the Chi-squared test or Fisher test was used across applicable groups. *p < 0.05, **p < 0.01, ***p < 0.001 compared with healthy control subjects. #p < 0.05, ##p < 0.01, ###p < 0.001 compared with T2- intermediate. §p < 0.05, §§p < 0.01, §§§p < 0.001 compared with T2- low.

Abbreviations: ACQ-5: Asthma Control Questionnaire - 5; BMI: body mass index; FeNO: Fractional concentration of exhaled nitric oxide; FEV1: Forced expiratory volume in 1 second; FVC: Forced vital capacity; ICS: inhaled corticosteroids; [IQR]: interquartile range; LABA: long-acting Beta2 agonist; LAMA: long-acting muscarinic antagonist; LTRA: leukotriene receptor antagonist; OCS: oral corticosteroids; SD: standard deviation.

# SUPPLEMENTARY FIGURES

## **Figure S1. S**ummary of study design

## Figure S2. Correlations between clinical characteristics in asthma.

(**A**) Correlation matrix of clinical variables. Spearman correlation matrix showing pairwise associations between clinical characteristics. Circle colour indicates the direction of correlation (red = positive, blue = negative), and circle size reflects the correlation strength. Stars indicate statistical significance of the correlation: p < 0.05: *, p < 0.01: **,p < 0.001: ***.

(**B**) Scatter plot of the correlation between fractional exhaled nitric oxide (FeNO) levels and blood eosinophil counts (cells × 10^9/L) in patients with asthma. Both axes are logarithmic. A linear regression line (red) with a 95% confidence interval (grey) is fitted to the data. (Spearman’s R = 0.45, p = 0.013), indicating a moderate, positive correlation between FeNO levels and blood eosinophil counts in this patient population.

## Figure S3. Heatmap of differentially expressed genes across asthma, healthy with ICS, and healthy control groups in bronchial biopsies.

The heatmap displays the expression levels of selected asthma signature genes across three groups: asthma patients (orange), healthy controls using inhaled corticosteroids (ICS; pink), and healthy controls not using ICS (grey). Each row represents one gene, and each column represents one individual sample. Gene expression values are z-score normalised across samples, with red indicating higher expression and blue indicating lower expression. Genes were selected based on established asthma signatures from Table 2.

Figure S4. Cell type composition of asthma and healthy controls**.**

**A**) Deconvolution analysis based on the Travaglini Lung Atlas comparing asthma *versus* healthy controls not receiving ICS. **B**) Deconvolution analysis based on the Travaglini Lung Atlas (B) comparing different phenotypes of asthma. Boxplots indicate median and interquartile ranges. Statistical significance was assessed using ANOVA.

## Figure S5. Type 2 (T2) and IL-17 signature expression across T2-high, -intermediate, and -low asthma, healthy controls.

**A**) T2 and IL-17 signature scores used IL-13–induced genes (POSTN, SERPINB2, and CLCA1) and IL-17–induced chemokines (CXCL1, CXCL2, CXCL3, IL8, and CSF3) to estimate Type 2 (T2) and IL-17 immune activity. Healthy controls were not receiving ICS. Signature scores were derived by averaging log2-transformed signature gene expression counts that have been normalised by sample library size, plus 1 (to accommodate genes with a normalised count of 0). **B**) The correlation between T2 and IL-17 signature scores in asthma.

**Figure S6.** Weighted gene co-expression network analysis (WGCNA) on bronchial biopsy differentially expressed genes in asthma.

(**A**) Clustering of module eigengenes based on their correlation. Modules with similar expression profiles across samples are grouped together, indicating higher-order relationships among co-expression modules.

(**B**) Clustering of module eigengenes based on their correlation. Modules with similar expression profiles across samples are grouped together, indicating higher-order relationships among co-expression modules.

(**C**) Gene clustering dendrogram based on topological overlap, with assigned module colours displayed below. Each branch corresponds to a gene cluster (module) identified by dynamic tree cutting, with colour assignments representing distinct modules.

(**D**) Modules were named according to their top significantly enriched Gene Ontology (GO) biological process term. Each cell displays the correlation coefficient (top) and the corresponding p-value (bottom, in parentheses). Blue indicates negative correlations, and red indicates positive correlations; more saturated colours represent stronger associations.

(**E**) Gene Ontology (GO) enrichment analysis of selected WGCNA modules. The dot plot shows representative GO biological process terms enriched in the selected modules. Dot size reflects the proportion of genes in each module associated with the GO term (GeneRatio), and dot colour represents the adjusted p-value. ME, module eigengene. For sex, we compare female versus male.

Figure S7. Log2 fold change of asthma versus health (no ICS) in RASP plotted against asthma versus health no ICS in U-BIOPRED. **A**) Biopsies, **B**) Brushes.

Figure S80. Log2 fold change of T2-high versus T2-low asthma in RASP plotted against T2-high versus T2-low asthma in U-BIOPRED. **A**) Biopsies, **B**) Brushes.

Figure S9. Log2 fold change of T2-high versus T2-intermediate asthma in RASP plotted against T2-high versus T2-low asthma in U-BIOPRED. **A**) Biopsies, **B**) Brushes
